# BCL6 is regulated by the MAPK/ELK1 axis and promotes *KRAS*-driven lung cancer

**DOI:** 10.1172/JCI161308

**Published:** 2022-11-15

**Authors:** Kun Li, Yanan Liu, Yi Ding, Zhengwei Zhang, Juanjuan Feng, Jiaxin Hu, Jiwei Chen, Zhengke Lian, Yiliang Chen, Kewen Hu, Zhi Chen, Zhenyu Cai, Mingyao Liu, Xiufeng Pang

**Affiliations:** 1 Changning Maternity and Infant Health Hospital and Shanghai Key Laboratory of Regulatory Biology and School of Life Sciences and; 2 Joint Translational Science and Technology Research Institute, East China Normal University, Shanghai, China.; 3 Cancer Institute, Fudan University Shanghai Cancer Center, Department of Oncology, Shanghai Medical College, Fudan University, Shanghai, China.; 4 Department of Biochemistry and Molecular Biology, School of Medicine, Tongji University, Shanghai, China.; 5 Medical Research Institute, Wuhan University, Wuhan, China.

**Keywords:** Oncology, Therapeutics, Lung cancer

## Abstract

Mutational activation of KRAS is a common oncogenic event in lung cancer, yet effective therapies are still lacking. Here, we identify B cell lymphoma 6 (BCL6) as a lynchpin in *KRAS*-driven lung cancer. BCL6 expression was increased upon KRAS activation in lung tumor tissue in mice and was positively correlated with the expression of KRAS-GTP, the active form of KRAS, in various human cancer cell lines. Moreover, BCL6 was highly expressed in human *KRAS*-mutant lung adenocarcinomas and was associated with poor patient survival. Mechanistically, the MAPK/ERK/ELK1 signaling axis downstream of mutant KRAS directly regulated BCL6 expression. BCL6 maintained the global expression of prereplication complex components; therefore, BCL6 inhibition induced stalling of the replication fork, leading to DNA damage and growth arrest in *KRAS*-mutant lung cancer cells. Importantly, BCL6-specific knockout in lungs significantly reduced the tumor burden and mortality in the LSL-*Kras*^G12D/+^ lung cancer mouse model. Likewise, pharmacological inhibition of BCL6 significantly impeded the growth of *KRAS*-mutant lung cancer cells both in vitro and in vivo. In summary, our findings reveal a crucial role of BCL6 in promoting *KRAS*-addicted lung cancer and suggest BCL6 as a therapeutic target for the treatment of this intractable disease.

## Introduction

Lung cancer is the leading cause of cancer incidence and mortality worldwide (1). The overall 5-year survival rate of lung cancer remains low(2). Previous molecular studies have identified several oncogenic drivers and promoted the development of satisfactory treatments for lung cancer driven by alterations in EGFR, ALK, RET, or ROS1 (3). However, few effective therapies are available for gain-of-function mutations in *KRAS*, which occur in approximately 25% of all lung cancer cases. Knowledge of KRAS protein structure, dynamics, and signal transduction remains unmet, largely preventing the development of specific inhibitors that target this oncogene directly or indirectly. The selective KRAS inhibitor (KRASi) sotorasib, which specifically targets KRAS(G12C) by creating a stable covalent bond with a mutant cysteine residue, has been approved by the US Food and Drug Administration as a second-line treatment for locally advanced or metastatic non–small cell lung cancer carrying the KRAS(G12C) mutation (4). However, drug resistance inevitably occurs during treatment (5, 6). Importantly, other frequently mutated forms of KRAS in lung cancer, such as KRAS(G12D) and KRAS(G12V), remain undruggable (7). Targeting downstream effectors of KRAS, such as MEK, is an alternative strategy to inhibit oncogenic KRAS signaling. However, the use of MEKi as a single agent or in combination with other clinical drugs has failed to demonstrate significant survival outcomes in *KRAS*-driven lung cancer, partly because of reactivation of the MAPK pathway component or reduced dominant intratumoral T cell clones (8, 9). Therefore, identification and further development of promising therapeutic strategies are urgently needed related to either novel KRAS pathway components or KRAS-related proteins that are synthetic lethal with mutant KRAS.

B cell lymphoma 6 (BCL6) was initially discovered as a protooncogene and is required for the formation of germinal center and development of B cells (10, 11). During clonal expansion and immunoglobulin affinity maturation, BCL6 enables B cells to tolerate DNA damage by regulating DNA damage–sensing genes (*ATR*, *TP53*, *ARF*, and others) (10). BCL6 also favors the survival and proliferation of germinal center B cells by modulating proliferation checkpoint genes (*CDKN1A, CDKN2A, PTEN*, and others) (12) and numerous target oncogenes (*MYC, BCL2, BMI1*, and others) (13, 14). Therefore, BCL6 has been characterized as a critical transcriptional repressor in germinal center. However, constitutive expression of BCL6 by chromosomal translocations or aberrant somatic mutations causes diffuse large B cell lymphoma (12). Current inhibitors either block the interaction between the BTB domain and correpressors or promote BCL6 degradation. These have shown antitumor effects against BCL6-addicted tumors (15, 16). Recent studies have implicated genomic amplification of the *BCL6* locus in certain solid tumors such as glioma, breast cancer, and ovarian cancer (17–19). Moreover, the combined inhibition of BCL6 and STAT3 synergistically defeats intratumoral heterogeneity in a subset of non–small cell lung cancers (20). We recently revealed that BCL6 enables solid tumor cells to evade genotoxic stress (21), and pharmacological inhibition of BCL6 enhances the sensitivity of *KRAS*-mutant lung cancer cells to clinical BETi (22). However, the biological function of BCL6 in *KRAS*-mutant lung tumorigenesis remains unclear. The fact that BCL6 functions as a protooncogene and is required for malignant transformation prompts us to speculate that BCL6 may be linked to oncogenic responses in lung cancer.

In this study, we demonstrated that the MAPK/ETS transcription factor ELK1 (MAPK/ELK1) signaling axis directly upregulated BCL6 expression in the context of KRAS mutational activation. Increased BCL6 expression enhanced the expression of prereplication complex (preRC) components, thereby maintaining cellular replication origins and cell cycle progression. Importantly, BCL6 was functionally required for *KRAS*-mutant lung tumorigenesis and tumor growth, as the genetic or pharmacological inhibition of BCL6 significantly impeded the growth of *KRAS*-mutant lung cancer in preclinical mouse models. Our findings provide a direct link between BCL6 and KRAS oncogenic signaling and highlight that BCL6 is an important target for the treatment of *KRAS*-driven lung cancer.

## Results

### Mutant KRAS promotes BCL6 expression.

**Figure 1:**
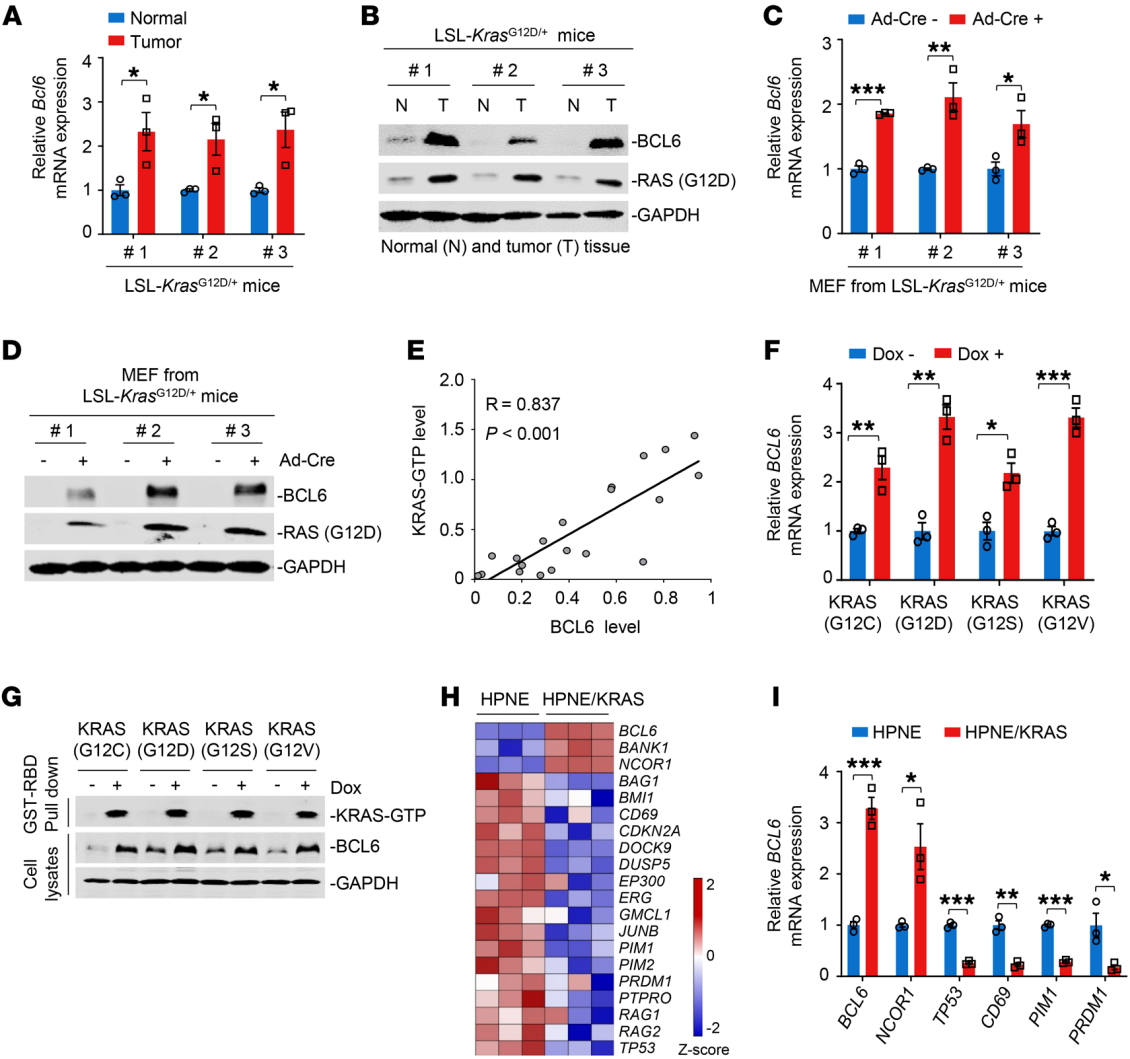
Mutant KRAS promotes BCL6 expression. (**A** and **B**) mRNA (**A**) and protein (**B**) expression levels of BCL6 in normal lung (N) and tumor tissue (T) from LSL-*Kras*^G12D/+^ mice (*n* = 3). LSL-*Kras*^G12D^ mice were infected with intranasal Ad-Cre for 16 weeks. (**C** and **D**) mRNA (**C**) and protein (**D**) expression levels of BCL6 in MEFs. MEFs were isolated from LSL-*Kras*^G12D^ mice and infected with Ad-Cre for 72 hours (*n* = 3). (**E**) Correlation analysis. Correlation between BCL6 and KRAS-GTP protein expression levels in different cancer cell lines (*n* = 20). KRAS-GTP levels were determined using GST-RBD, the GST-fusion of the RAS binding domain of C-RAF, to pulldown active GTP-bound RAS from cellular lysates by glutathione beads. R, Pearson’s correlation coefficient. (**F** and **G**) KRAS mutant variants upregulated BCL6 expression at the mRNA (**F**) and protein (**G**) levels. 293T cells were transfected with different doxycycline-inducible G12 KRAS mutant variants: pLVX-TetOne-KRAS(G12C), pLVX-TetOne-KRAS(G12D), pLVX-TetOne-KRAS(G12S), pLVX-TetOne-KRAS(G12V). Transfected cells were then treated with or without 2 μM doxycycline (Dox) for 96 hours. KRAS activity was determined using GST-RAF-RBD to pulldown GTP-KRAS from cell lysates. (**H**) Heatmap showing differentially expressed BCL6 target genes (*P* < 0.05) in HPNE and HPNE/KRAS cells (*n* = 3). *z* score was calculated based on counts of exon model per million mapped reads. (**I**) The mRNA expression levels of BCL6 target gene in HPNE and HPNE/KRAS cells (*n* = 3). Data in **A**, **C**, **F**, and **I** are expressed as mean ± SEM of 3 technical replicates, representative of 3 independent experiments. *P < 0.05, **P < 0.01, ***P < 0.001, determined via unpaired 2-tailed Student’s *t* test. The immunoblots in **B**, **D**, and **G** were contemporaneous and run in parallel from the same biological replicate.

**Figure 2:**
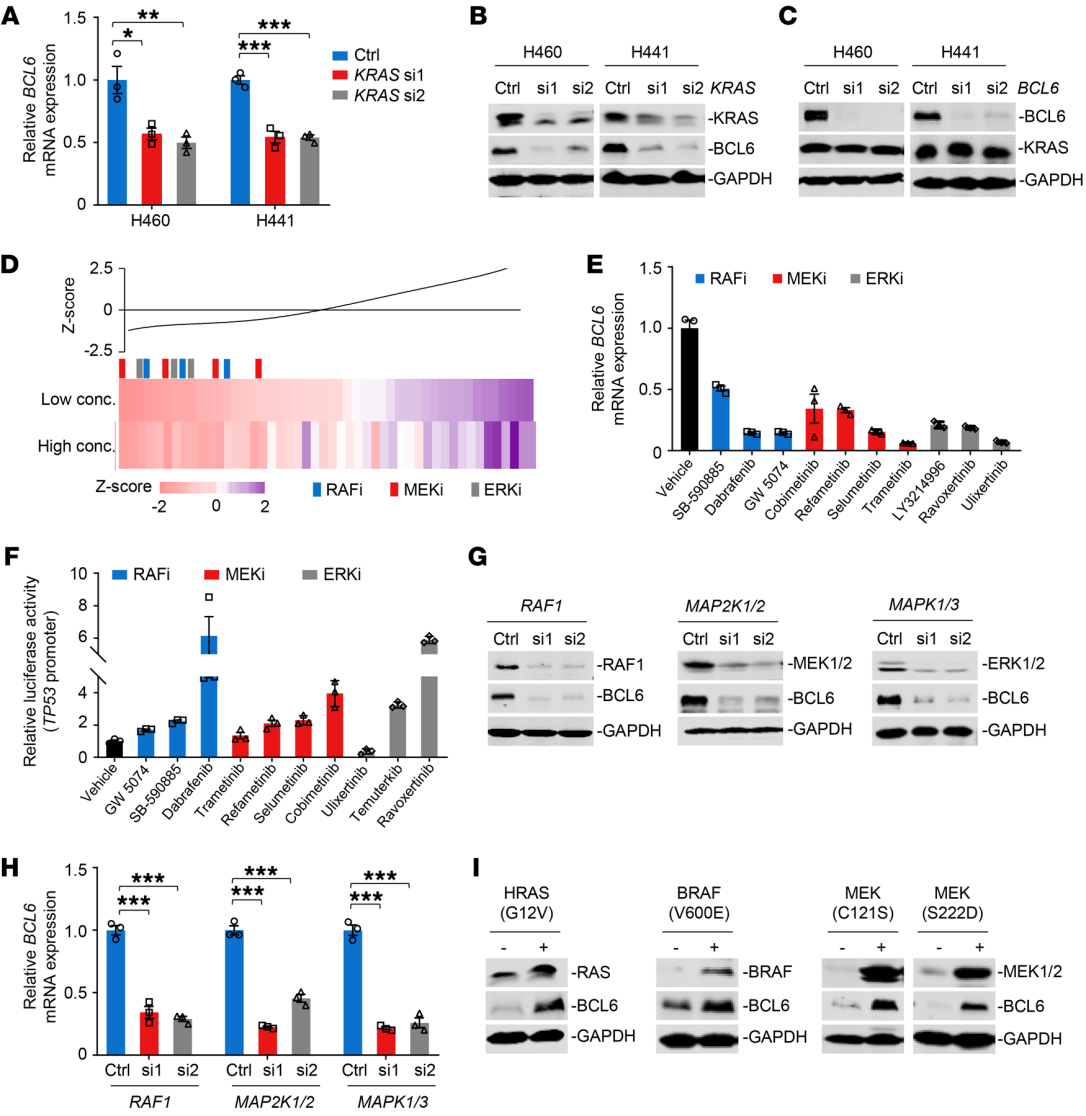
The MAPK/ERK signaling axis contributes to BCL6 expression. (**A** and **B**) *KRAS* knockdown downregulated BCL6 mRNA (**A**) and protein (**B**) expression. Cells were transfected with 20 nM siRNAs targeting *KRAS* for 48 hours. (**C**) *BCL6* silencing did not affect KRAS expression. (**D**) The MAPK/ERK pathway inhibitors suppressed BCL6 expression. H460 cells were treated with a small-molecule library consisting of 48 compounds at their respective IC_50_s (Low conc.) or double IC_50_s (High conc.) for 48 hours. BCL6 levels detected by immunoblotting analysis in compound-treated cells were normalized to those in DMSO-treated cells. The readout of each compound was analyzed into a *z* score as a line graph and as a gradient colored bar. Colored vertical bars indicate the inhibitor class, and the black bar indicates the mean. (**E**) The MAPK/ERK pathway inhibitors downregulated *BCL6* mRNA expression. H460 cells were treated with the indicated inhibitors at a concentration of their respective IC_50_s for 48 hours. (**F**) The MAPK/ERK pathway inhibitors increased the *TP53*-pGL3 reporter activity. H460 cells were transiently transfected with the *TP53*-pGL3 reporter plasmid before treatment. The readout of luciferase values in treated groups was normalized to that in the DMSO-only group. (**G** and **H**) Knockdown of *RAF1*, *MAP2K1/2*, and *MAPK1/3* inhibited BCL6 protein (**G**) and mRNA (**H**) expression levels in H460 cells. (**I**) Constitutively active variants upregulated BCL6 expression in 293T cells. Data in **A**, **E**, **F**, and **H** are expressed as mean ± SEM of 3 technical replicates, representative of 3 independent experiments with similar results. Statistical analyses in **A** and **H** were performed using 1-way ANOVA with Tukey’s multiple comparison test, *P < 0.05, **P < 0.01, ***P < 0.001. The immunoblots in **B**, **C**, **G**, and **I** were contemporaneous and run in parallel from the same biological replicate, representative of 3 independent experiments.

The deregulated expression of BCL6, a master regulator of germinal center formation, is often linked to lymphomagenesis. Sustained BCL6 expression is also required to maintain malignant phenotypes in certain solid tumors (10). However, the role of BCL6 in lung cancer remains to be elucidated. Given that BCL6 elicits transcriptional activity and directly regulates multiple target genes, we hypothesized that BCL6 plays a role in lung cancer. Therefore, we determined BCL6 expression in an LSL-*Kras*^G12D/+^ mouse model of lung cancer. Strikingly, we observed a significant tumor burden accompanied by high BCL6 expression in mouse lung lesions 4 months after intranasal administration of adenovirus-Cre (Ad-Cre) (Supplemental Figure 1, A and B; supplemental material available online with this article; https://doi.org/10.1172/JCI161308DS1). Quantitative PCR (qPCR) and immunoblot analysis further showed that the mRNA and protein expression levels of BCL6 in mouse lung tumors harboring the KRAS(G12D) mutation were significantly higher than those in adjacent normal lung tissue (Figure 1, A and B). Similar results of BCL6 induction were observed in mouse embryonic fibroblasts (MEFs) isolated from LSL-*Kras*^G12D/+^ mice (Figure 1, C and D) and 2 pairs of engineered KRAS isogenic cell lines (Supplemental Figure 1C) upon KRAS mutational activation. Next, we examined BCL6 and KRAS-GTP protein expression in various human lung, colorectal, and pancreatic cancer cell lines and observed a positive correlation (Figure 1E and Supplemental Figure 1D). To investigate the regulatory link between mutant KRAS and BCL6, we overexpressed different doxycycline-inducible G12 KRAS mutant variants (G12C, G12D, G12S, and G12V) in 293T cells, all of which commonly occur in human lung cancer. The mRNA and protein expression levels of BCL6 were robustly increased in the presence of activating KRAS mutations (Figure 1, F and G), reinforcing the general role of BCL6 in response to different KRAS mutant variants. We further showed that increased BCL6 abundance was observed in the nuclei of *KRAS*-mutant cells and was not due to altered protein stability (Supplemental Figure 1, E and F).

BCL6 is a known transcriptional repressor that regulates hundreds of target genes by recruiting different chromatin-modifying corepressor complexes (14, 23). We next explored whether the KRAS oncogene enhances the global transcription of BCL6 target genes by performing RNA sequencing (RNA-Seq) in *KRAS* WT and mutant cells. Although some functions of BCL6 appear to be conserved between normal and malignant cells, BCL6 transcriptional programs would be altered in cells with different genomic profiles or in specific biological contexts (14, 23). Given that BCL6 target gene sets have not been fully defined in solid tumors, we thus used comparative BCL6 target gene selection to identify the genes that were differentially expressed between KRAS WT and mutant cells. Our data revealed that the core of BCL6 target genes, including *BCL6*, was affected by the inducible activation of KRAS in engineered isogenic cells (Figure 1H) and MEFs (Supplemental Figure 1G), supporting the notion that oncogenic KRAS regulates BCL6 signaling. The altered expression of representative, differentially expressed target genes, such as *BCL6*, *NCOR1*, *TP53*, *CD69*, *PIM1*, and *PRDM1*, was further confirmed by qPCR analysis (Figure 1I and Supplemental Figure 1H). These results indicate that KRAS mutational activation leads to sustained expression of BCL6 and its target genes.

### The MAPK/ERK signaling axis contributes to BCL6 expression.

Next, we explored how the oncogenic KRAS protein promotes BCL6 expression using genetic and chemical approaches. We genetically silenced *KRAS* and *BCL6* using siRNAs in H460 and H441 cells. Our results demonstrated that *KRAS* deficiency abolished both the mRNA and protein expression of BCL6 (Figure 2, A and B), whereas *BCL6* silencing did not affect KRAS expression (Figure 2C), indicating that BCL6 was downstream of KRAS. To further delineate which KRAS effector pathway contributes to BCL6 expression, we employed a small library consisting of 48 small-molecule inhibitors targeting crucial KRAS effectors, including MAPK, phosphatidyl inositol 3-kinase, and Ral pathway components (Supplemental Table 1). We observed decreased BCL6 protein expression in treated H460 cells and demonstrated that the MEKi selumetinib was the most effective hit. Intriguingly, 20 top-ranked hits from this screen were all MAPK/ERK pathway inhibitors, including RAFi, MEKi, and ERKi (Figure 2D), indicating the positive regulation of the MAPK/ERK axis on BCL6 expression. Moreover, BCL6 expression was reduced at the transcriptional level (Figure 2E).

BCL6 has been reported to directly bind to 2 specific sites within the *TP53* promoter region and further suppresses its transcription (24). Next, we performed a *TP53* reporter assay (Supplemental Figure 2A) and examined the effects of the MAPK/ERK pathway inhibitors on BCL6-mediated *TP53* transcription. Luciferase reporter assays showed that most MAPK/ERK pathway inhibitors led to an increase in reporter activity (Figure 2F), reinforcing an essential role of the MAPK pathway in regulating BCL6 biological activity. In line with this, siRNAs targeting *RAF1*, *MAP2K1/2*, and *MAPK1/3* dramatically inhibited BCL6 protein and mRNA levels in H460 (Figure 2, G and H) and H441 cells (Supplemental Figure 2, B and C), respectively. In contrast, constitutively active variants of HRAS(G12V), BRAF(V600E), MEK(C121S), or MEK(S222D) significantly augmented BCL6 expression (Figure 2I). Analysis of human lung cancer data sets derived from The Cancer Genome Atlas (TCGA) showed that *KRAS*, *RAF1*, *MAP2K1*, *MAP2K2*, *MAPK3*, and *MAPK1* expression levels were positively correlated with the *BCL6* expression level (Supplemental Figure 3). Collectively, our results suggest that the MAPK/ERK signaling axis promotes BCL6 expression in *KRAS*-mutant lung cancer cells.

### ELK1 directly binds to BCL6 promoter and promotes its expression.

**Figure 3:**
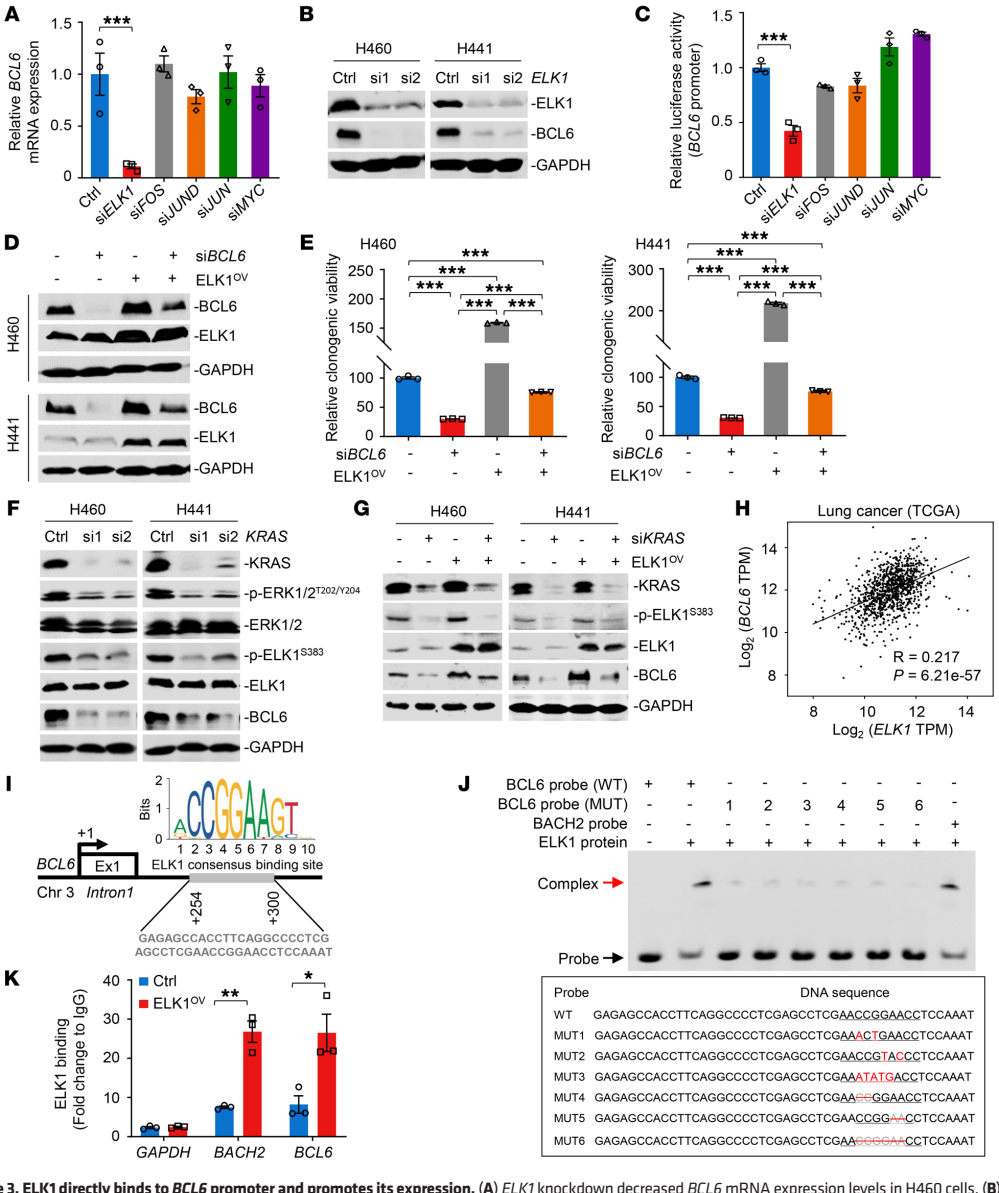
ELK1 directly binds to *BCL6* promoter and promotes its expression. (**A**) *ELK1* knockdown decreased *BCL6* mRNA expression levels in H460 cells. (**B**) *ELK1* knockdown decreased *BCL6* protein levels. (**C**) *ELK1* silencing suppressed *BCL6* promoter activity. H460 cells were cotransfected with *BCL6*-pGL3 and indicated siRNAs for 48 hours. (**D**) Exogenous transduction of ELK1 (ELK1^OV^) in *BCL6*-depleted cells restored BCL6 expression. (**E**) Ectopically expressed ELK1 increased the clonogenic growth of *BCL6*-depleted cells. The relative clonogenic viability was calculated by setting the untreated group as 100%. (**F**) *KRAS* knockdown reduced pERK1/2^T202/Y204^, pELK1^S383^, and BCL6 levels. (**G**) Exogenous transduction of ELK1 (ELK1^OV^) in *KRAS*-depleted cells restored BCL6 expression. (**H**) Scatterplot showing a positive correlation of *BCL6* mRNA expression levels with *ELK1* mRNA expression levels in human lung cancer data sets derived from the TCGA. *n* = 1145. R, Pearson’s correlation coefficient. (**I**) Human *BCL6* locus containing ELK1 consensus binding site (Jaspar prediction) in exon1A region. The transcription start site is indicted as +1. Exon 1, Ex1. (**J**) Electrophoretic mobility shift assay showing the binding complex of ELK1 protein and indicated probes, as indicated by red and black arrows, respectively (*top*). The sequence of BCL6 mutant probes (MUT1–MUT6) are shown (*bottom*). The mutated bases are indicted in red, and base depletion are indicated in gray with a red line-through. (**K**) ChIP-qPCR data showing enrichment of ELK1 binding to the *BCL6* promoter. The fold change of ELK1 binding is shown. Data in **A**, **C**, **E**, and **K** are expressed as mean ± SEM of 3 technical replicates. Statistical analyses in **A**, **C**, and **E** were performed using 1-way ANOVA with Tukey’s multiple comparison test, and in **K** using unpaired 2-tailed Student’s *t* test, *P < 0.05, **P < 0.01, ***P < 0.001. The immunoblots in **B**, **D**, **F**, and **G** were contemporaneous and run in parallel from the same biological replicate.

Our results showed that mutant KRAS activated BCL6 expression through the MAPK/ERK signaling axis at the transcriptional level. Next, we characterized the mechanisms underlying this regulation. ELK1, c-Fos, JunD, c-Jun, and c-Myc have been reported to be critical transcription factors involved in the MAPK/ERK signaling cascade (25). Therefore, we investigated their effects on BCL6 expression after efficient silencing (Supplemental Figure 4). Surprisingly, we found that *ELK1* knockdown significantly decreased BCL6 expression at both the mRNA and protein levels in H460 cells (Figure 3, A and B). Data from luciferase reporter assays further showed that the *BCL6* promoter activity was specifically suppressed after *ELK1* silencing (Figure 3C). Notably, ectopic expression of ELK1 in *BCL6*-depleted cells restored BCL6 expression (Figure 3D), suggesting that BCL6 is dependent on ELK1 regulation. Moreover, *BCL6* depletion decreased the clonogenic growth of H460 and H441 cells, whereas this effect was apparently reversed by simultaneous ELK1 overexpression (Figure 3E). To further validate the modulation of BCL6 expression by the KRAS/MAPK/ELK1 cascade, we genetically silenced *KRAS* and observed a potent and coincident reduction in p-ERK1/2, p-ELK1, and BCL6 expression (Figure 3F); additionally, ectopic expression of ELK1 in *KRAS*-depleted cells apparently restored BCL6 abundance (Figure 3G). Analysis of TCGA database showed that *ELK1* expression levels were positively correlated with *BCL6* expression levels in human lung cancers (Figure 3H). Collectively, these data suggest that the MAPK/ERK/ELK1 signaling axis promotes BCL6 expression.

A relatively short region in the first noncoding exon1A of BCL6 (+254 to +300) has been characterized as its transcriptional binding site and is associated with high levels of *BCL6* transcriptional activity (26, 27). Thus, we performed in silico motif analysis using the JASPAR database (28) and identified an ELK1-binding motif in BCL6 noncoding exon1A region (Figure 3I). As the transcriptional repressor BACH2 is directly controlled by ELK1 to sustain the T follicular helper cell phenotype and function (29), we used the reported BACH2 binding motif as the control. To show the direct binding of ELK1 to the BCL6 exon1A region, we performed electrophoretic mobility shift assays, where a 47-bp probe for the BCL6 exon1A region containing the ELK1-binding motif (WT probe) was incubated with purified ELK1 protein. Notably, the DNA-protein complex-shifted band was clearly observed in the presence of the BCL6 WT probe (Figure 3J). In contrast, BCL6 probes with mutated bases or base depletion in CCGGAA motif reduced the binding activity to the ELK1 protein, suggesting the CCGGAA motif as the critical binding site. Using chromatin immunoprecipitation followed by qPCR analysis, we further confirmed the occupancy of ELK1 on the BCL6 exon1A region in H460 cells (Figure 3K). Altogether, our results reveal a direct binding of ELK1 to BCL6, which is essential for *BCL6* transcription.

### BCL6 inhibition impedes the growth of KRAS-mutant cancer cells in vitro.

**Figure 4:**
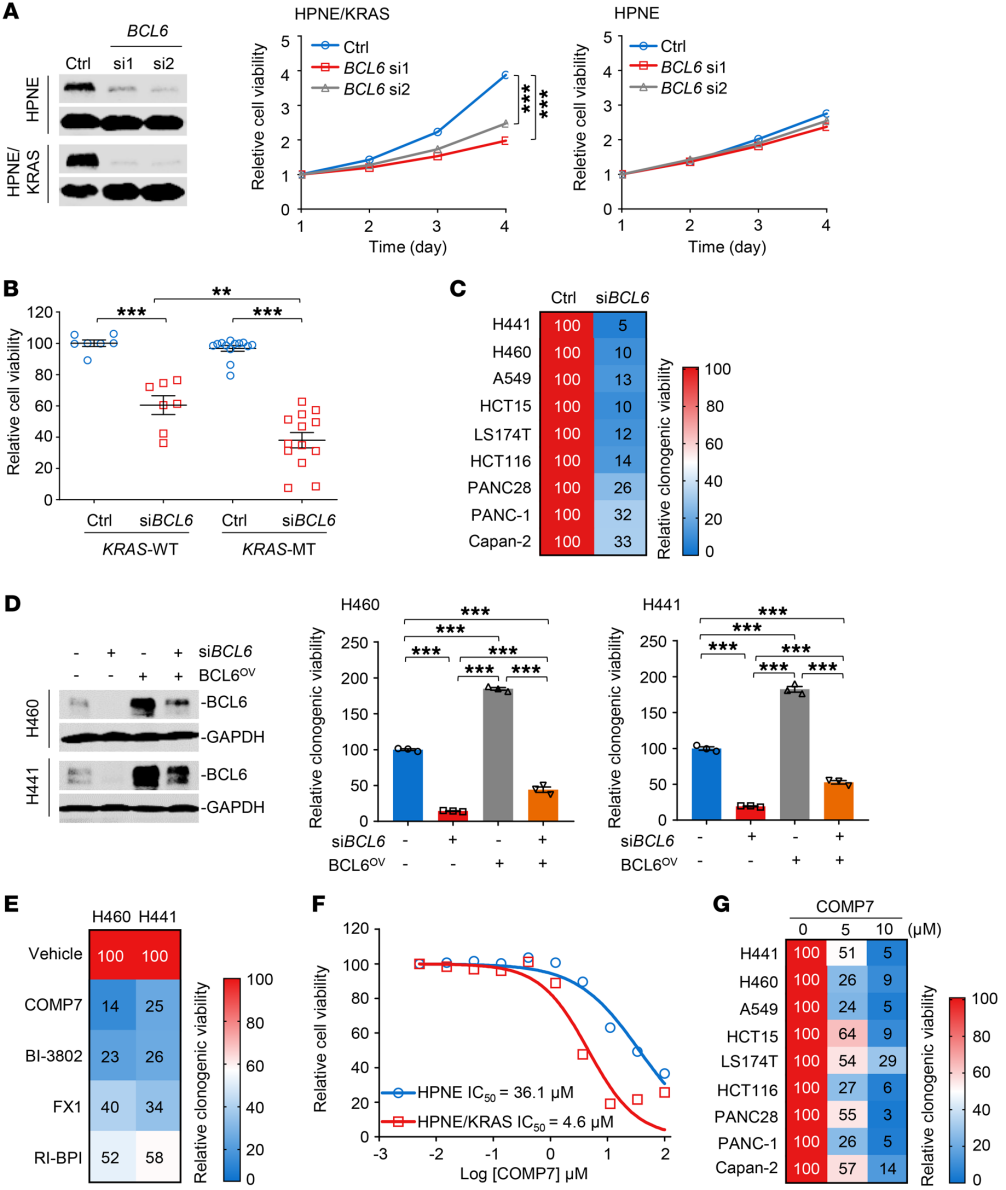
BCL6 inhibition impedes the growth of *KRAS*-mutant cancer cells in vitro. (**A**) Effects of *BCL6* knockdown on cell survival. The knockdown efficiency of si*BCL6* was examined (left) and cell viability was measured using a Cell Counting Kit-8 (CCK-8) (right). Relative cell viability (normalized to day 0) is plotted. (**B**) *BCL6* depletion led to selective cytotoxic effects toward *KRAS*-mutant cancer cell lines. Thirteen *KRAS*-mutant and 7 *KRAS*-WT cancer cell lines were transfected with si*BCL6* or a scrambled siRNA control. Cell viability was measured 72 hours after transfection using a CCK-8 assay kit. Relative cell viability is shown by setting the untreated control as 100%. (**C**) The colony-formation ability of indicated cell lines after transfection with si*BCL6* or siControl. The relative clonogenic viability is calculated by normalizing the untreated group as 100%. (**D**) Exogenous transduction of BCL6 (BCL6^OV^) reduced *BCL6* knockdown–mediated cytotoxicity. BCL6 expression (left) and the relative cell viability of cultured colonies (right) are shown. (**E**) The colony-formation ability of indicated cell lines after treatment with BCL6i. The relative viability of cultured colonies is calculated by normalizing the untreated group as 100%. (**F**) The inhibitory effects of COMP7 on HPNE and HPNE/KRAS cells. Cells were treated with COMP7 at gradient concentrations for 72 hours. (**G**) COMP 7 suppressed the clonogenic growth of various *KRAS*-mutant cancer cell lines. Cells were treated with 5 μM or 10 μM COMP7 for 7 days. Data in **A**, **B**, **D**, and **F** are expressed as mean ± SEM of 3 technical replicates. Statistical analyses in **A**, **B**, and **D** were performed using 1-way ANOVA with Tukey’s multiple comparison test, *P < 0.05, **P < 0.01, ***P < 0.001. The immunoblots in **A** and **D** were contemporaneous and run in parallel from the same biological replicate, representative of at least 3 independent experiments.

Given that the KRAS/MAPK/ELK1 axis directly regulates BCL6 expression, we next investigated whether activated BCL6 promoted *KRAS*-mutant lung cancer. First, we investigated the effects of *BCL6* knockdown on tumor cell growth in vitro. We genetically silenced *BCL6* in the HPNE cell pair (Figure 4A) and found that *BCL6* silencing elicited cytotoxic effects selectively toward *KRAS*-mutant cells, whereas their WT counterparts were minimally affected (Figure 4A). We used a panel of human lung, colorectal, and pancreatic cancer cell lines and examined the effects of *BCL6* knockdown on their survival. Although *BCL6* depletion led to growth arrest in all tested cell lines, *KRAS*-mutant cancer cells were more susceptible to BCL6 inhibition than WT cancer cells (Figure 4B and Supplemental Figure 5A). Furthermore, *BCL6* knockdown dramatically reduced the clonogenic growth of various *KRAS*-mutant cancer cell lines (Figure 4C and Supplemental Figure 5B). The reduced growth of H460 and H441 cells following *BCL6* depletion could be rescued by BCL6 overexpression, suggesting a BCL6-dependent effect (Figure 4D).

To test whether BCL6 induction creates an actionable vulnerability, we treated *KRAS*-mutant cancer cells with BCL6 chemical inhibitors (15, 16, 30, 31). Our results showed that the 4 reported BCL6i elicited potent cytotoxicity in multiple *KRAS*-mutant cancer cell lines (Figure 4E and Supplemental Figure 5C). When noted, COMP7, which targets the BTB/POZ domain of BCL6 and prevents partner binding (15), achieved the best therapeutic efficacy. BI-3802 also displayed antiproliferative activity in our study; however, its poor bioavailability limits its application in animal studies (32). Therefore, we selected COMP7 for further investigation. In agreement with the results of *BCL6* genetic silencing, treatment with COMP7 resulted in much lower IC_50_ values in *KRAS*-mutant cells than in WT cells, suggesting that BCL6 inhibition creates de novo vulnerability specific to *KRAS*-mutant cells (Figure 4F). Moreover, COMP7 treatment attenuated the clonogenic growth of various *KRAS*-mutant cancer cell lines, eliciting a general inhibitory effect (Figure 4G and Supplemental Figure 5D).

Single targeting of MEK was insufficient to inhibit *KRAS*-driven lung cancer, partly because of sustained ERK reactivation (33). Given that the MAPK/ERK/ELK1 axis regulates BCL6 expression, we additionally explored whether BCL6 pharmacological inhibitors, such as COMP7, in combination with MEKi, could lead to more potent tumor inhibition than single-agent treatment. To this end, we combined COMP7 with the MEKi trametinib and examined the combined effects on the viability of H460 and H441 cells by analyzing the drug combination index (CI). Our results showed that most CI values at diluted drug concentrations were less than 1 (Supplemental Figure 6), indicating a synergistic effect of dual targeting of MEK and BCL6.

### BCL6 inhibition results in replication fork stalling and DNA damage.

**Figure 5:**
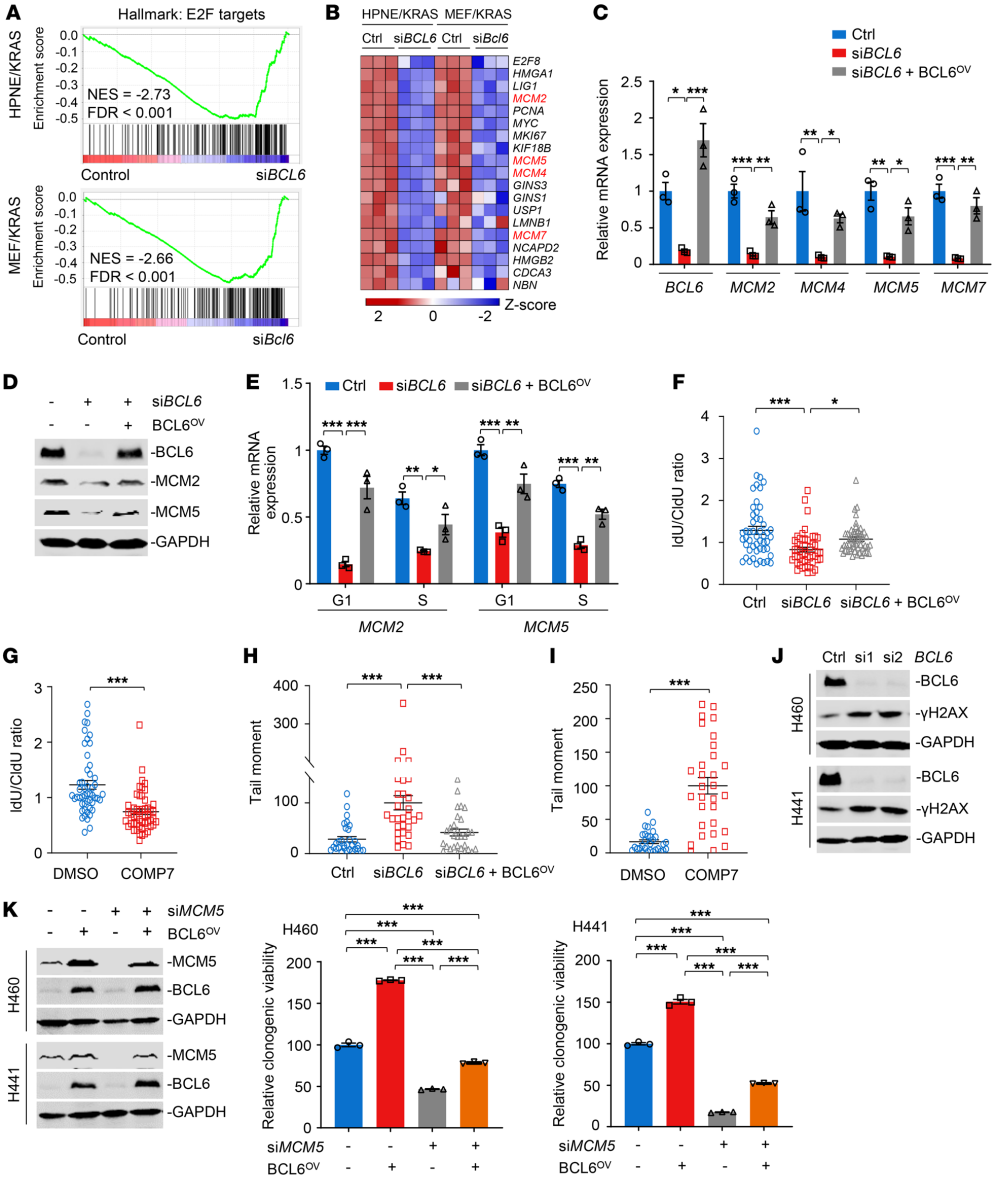
BCL6 inhibition results in replication fork stalling and DNA damage. (**A**) Gene set enrichment analysis of E2F targets in HPNE/KRAS (top) and MEF/KRAS cells (bottom). *n* = 3; NES, normalized enrichment score. (**B**) Heatmap showing E2F target gene expression in HPNE/KRAS and MEF/KRAS cells after *BCL6* knockdown. *z* score was calculated based on counts of exon model per million mapped reads. *n* = 3. (**C** and **D**) Exogenous transduction of BCL6 (BCL6^OV^) restored expression of preRC genes in *BCL6*-depleted cells. H460 cells were transfected with *BCL6* siRNAs alone or in combination with pcDNA 3.1-BCL6 vector. (**E**) *MCM2* and *MCM5* mRNA expression of sorted H460 cells. (**F** and **G**) DNA fiber analysis (*n* = 50 fibers). H460 cells were treated with genetic (**F**) or pharmacological approaches (10 μM COMP7) (**G**) Idu/CIdU ratio indicates red to green ratio. (**H** and **I**) Alkaline comet assays in H460 cells. The tail moment was defined as percentage of tail DNA × tail length using the Casp software. *n* = 30 cells. (**J**) *BCL6* knockdown increased γ-H2AX expression. (**K**) BCL6 overexpression reduced *MCM5* knockdown–mediated cytotoxicity. BCL6 and MCM5 expression (left) and the relative viability of cultured colonies (right) are shown. Data in **C**, **E**, **F**, **G**, **H**, **I**, and **K** were expressed as mean ± SEM Statistical analyses in **C**, **E**, **F**, **H**, and **K** were performed using 1-way ANOVA with Tukey’s multiple comparison test, and in **G** and **I** using unpaired 2-tailed Student’s *t* test, *P < 0.05, **P < 0.01, ***P < 0.001. The immunoblots in **D**, **J**, and **K** were contemporaneous and run in parallel from the same biological replicate, representative of at least 3 independent experiments.

Thereafter, we investigated the underlying mechanisms by which BCL6 inhibition blocked *KRAS*-mutant cancer growth. Genome-wide RNA-Seq was performed in HPNE/KRAS cells and MEF isolated from LSL-*Kras*^G12D^ mice (MEF/KRAS) upon *BCL6* efficient silencing. We analyzed the enriched pathways of the differentially expressed genes between *BCL6* silencing and the control groups. Our results showed that sets associated with cell cycle–related biological processes, such as E2F targets and MYC targets, were enriched and were in the leading rank of altered pathways (Supplemental Figure 7). Notably, the cell-cycle-promoting E2F target pathway was significantly downregulated after BCL6 silencing in both of the cell lines (Figure 5A). A heatmap of shared E2F target signature genes across HPNE/KRAS and MEF/KRAS cells showed significantly decreased expression of preRC components (Figure 5B), including hexameric minichromosome maintenance (MCM) 2, 4, 5, and 7, which form the core of replicative helicase at replication forks (34). Our qPCR results confirmed that *BCL6* depletion significantly reduced the expression of *MCM2*, *MCM4*, *MCM5*, and *MCM7*, whereas ectopic BCL6 expression in *BCL6*-knockdown cells largely reversed these effects (Figure 5C). Immunoblotting analysis showed similar results (Figure 5D).

PreRC proteins bind to DNA in the G1 phase and mark all potential replication origins, a subset of which is activated in well-defined steps during the S phase (35). Since *BCL6* silencing suppressed preRC expression, we deduced that *BCL6* knockdown might reduce the binding of preRC components to DNA, leading to replication fork stalling. To test this possibility, we analyzed the cell cycle progression in *KRAS*-mutant lung cancer cells after *BCL6* depletion alone or in combination with BCL6 reconstitution. As expected, *MCM2* and *MCM5* mRNA expression were significantly suppressed by *BCL6* silencing in both G1 and S phases, and an inverse effect was observed after BCL6 overexpression (Figure 5E). Furthermore, both genetic and chemical inhibition of BCL6 caused a significant decrease in DNA replication, as evidenced by DNA fiber analysis (Figure 5, F and G and Supplemental Figure 8, A and B).

MCM2–7 complexes are loaded onto DNA to license replication origins for use in the upcoming S phase. Inhibition of MCM loading is sufficient to induce DNA damage in eukaryotic cells (36, 37). Next, we performed alkaline comet assays to measure cellular DNA integrity after treatment. Our results demonstrated that *BCL6* silencing significantly increased DNA strand breaks, whereas this could be significantly decreased by BCL6 overexpression (Figure 5H and Supplemental Figure 8C). Consistently, the BCL6i COMP7 showed similar effects (Figure 5I and Supplemental Figure 8D). Meanwhile, the expression of γ-H2AX, a sensitive marker of DNA damage, increased after *BCL6* depletion in both H460 and H441 cells (Figure 5J). Importantly, the molecular regulation of BCL6 on preRC genes, such as *MCM5* was functional and linked to clonogenic growth of *KRAS*-mutant lung cancer cells (Figure 5K). It has reported that BCL6 overexpression attenuated while BCL6 knockdown enhanced the production of ROS levels in human vascular smooth muscle cells (38). As *KRAS*-mutated tumors are known to have high ROS levels and are more vulnerable to oxidative stress (25), it is possible that, in addition to DNA-replication regulation, the controlling effects of BCL6 on ROS levels might also contribute to the tumor-promoting effects of BCL6 in *KRAS*-mutant cancer cells. To test this possibility, we examined ROS levels upon genetic or pharmacological interference with BCL6 in H460 cells. Our results showed that BCL6 inhibition exerted little effect on ROS production in *KRAS*-mutant lung cancer cells (Supplemental Figure 9), thus excluding the role of ROS in BCL6-mediated tumor-promoting action in the context of KRAS mutational activation. Taken together, these data suggest preRC proteins as downstream targets of BCL6. Targeted inhibition of BCL6, therefore, leads to replication fork stalling, DNA damage, and ultimately, to growth arrest.

### Lung-specific ablation of BCL6 inhibits KRAS-driven lung tumorigenesis.

**Figure 6:**
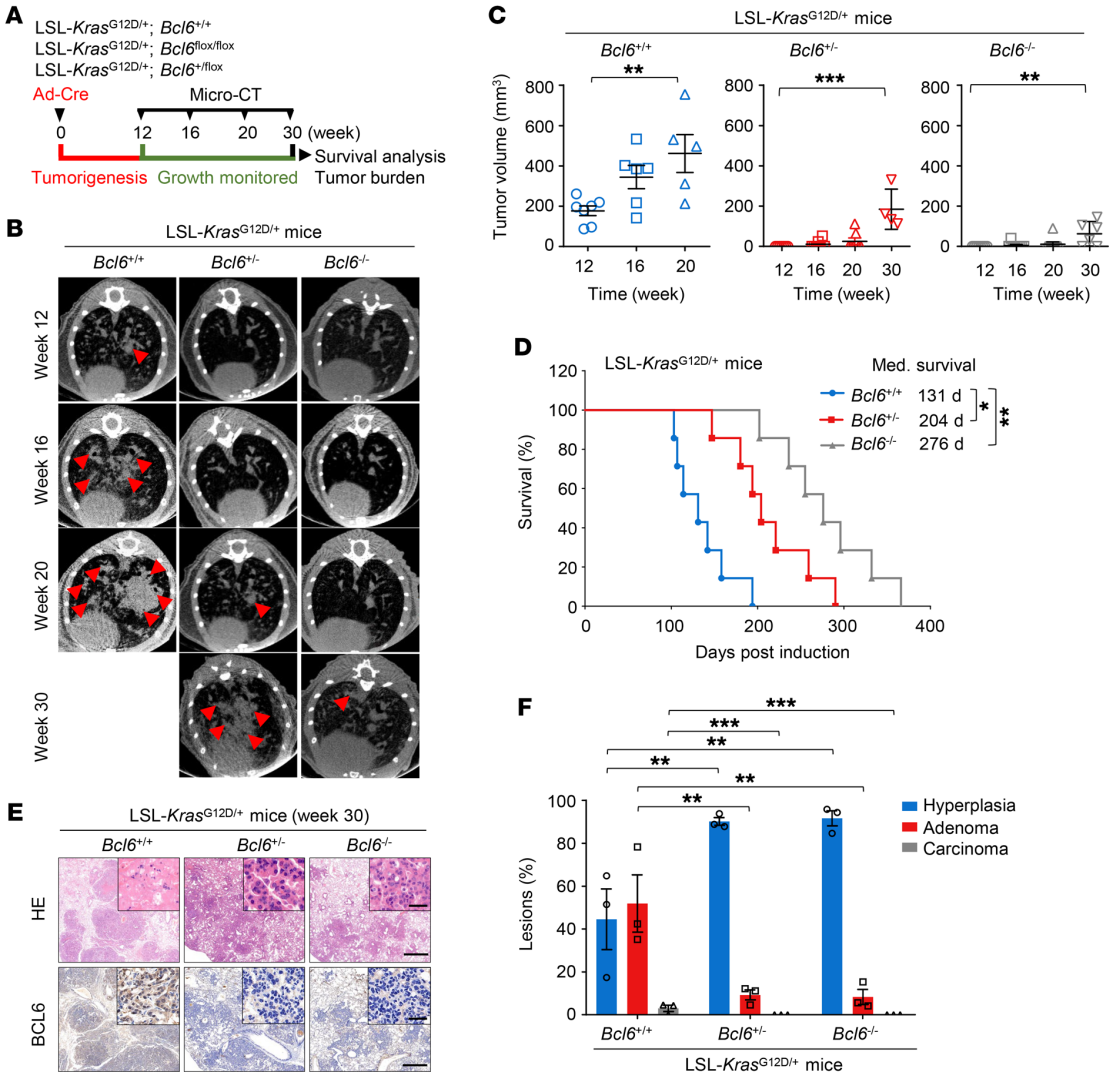
Lung specific ablation of BCL6 inhibits *KRAS*-driven lung tumorigenesis. (**A**) Schematic of experimental design. (**B**) Representative images of tumor from LSL-*Kras*^G12D/+^ mice: *Bcl6*^+/+^, *Bcl6*^+/–^, and *Bcl6*^–/–^. Micro-CT images showing axial planes of mouse lung. *n* = 7 per group. Tumor areas are indicated by red arrows. (**C**) Tumor volume of LSL-*Kras*^G12D/+^ mice: *Bcl6*^+/+^, *Bcl6*^+/–^, and *Bcl6*^–/–^. Quantification from micro-CT images represented in **B**. (**D**) Kaplan–Meier survival curves for LSL-*Kras*^G12D/+^ mice: *Bcl6*^+/+^, *Bcl6*^+/–^, and *Bcl6*^–/–^. *n* = 7 per group. (**E**) Representative H&E and IHC staining of lung from moribund LSL-*Kras*^G12D/+^ mice: *Bcl6*^+/+^, *Bcl6*^+/–^, and *Bcl6*^–/–^. *n* = 3 per group. Scale bars: 1 mm (inset), 50 μm (bottom). (**F**) Lesion classification of moribund LSL-*Kras*^G12D/+^ mice: *Bcl6*^+/+^, *Bcl6*^+/–^, and *Bcl6*^–/–^. *n* = 3 per group. Data in **C** and **F** are expressed as mean ± SEM of 3 technical replicates. Statistical analyses in **C** and **F** were performed using 1-way ANOVA with Tukey’s multiple comparison test, and in **D** using log-rank test, *P < 0.05, **P < 0.01, ***P < 0.001.

To assess the role of BCL6 in tumorigenesis and tumor growth in *KRAS*-driven lung cancer, we generated an engineered mouse model harboring the following alleles: (a) WT alleles of *Bcl6* with LSL-*Kras*^G12D/+^; (b) heterozygous floxed alleles for *Bcl6* with LSL-*Kras*^G12D/+^; and (c) homozygous floxed alleles for *Bcl6* with LSL-*Kras*^G12D/+^. The development of *Kras*-driven lung cancer in mice was monitored by noninvasive microcomputed tomography, as indicated in the experimental scheme (Figure 6A). According to previously published studies (39, 40), LSL-*Kras*^G12D/+^ mice developed lung adenomas 12 weeks after infection with Ad-Cre. Strikingly, our histological analysis showed that *Kras* activation with heterozygous or homozygous *Bcl6* loss in lung epithelium was devoid of any hyperplasia or adenomas and even retained normal histology 12 weeks after Ad-Cre administration (Supplemental Figure 10). LSL-*Kras*^G12D/+^ mice developed lung adenomas and highly variable tumor latencies 20 weeks after infection, with a median survival of 131 days, whereas *Bcl6* loss via heterozygous and homozygous recombination of floxed alleles significantly impeded tumor progression and prolonged mouse survival, with a median survival of 204 days and 276 days, respectively (Figure 6, B–D). Histopathological analysis of lung tumors from moribund autochthonous mice revealed that the mice harboring *Bcl6* floxed alleles displayed a significant decrease in the proportion of higher-grade adenomas or carcinomas (Figure 6, E and F). Altogether, our data suggest that BCL6 is functionally required for *KRAS*-driven lung tumorigenesis.

### Chemical inhibition of BCL6 impedes KRAS-mutant lung tumor growth in vivo.

**Figure 7:**
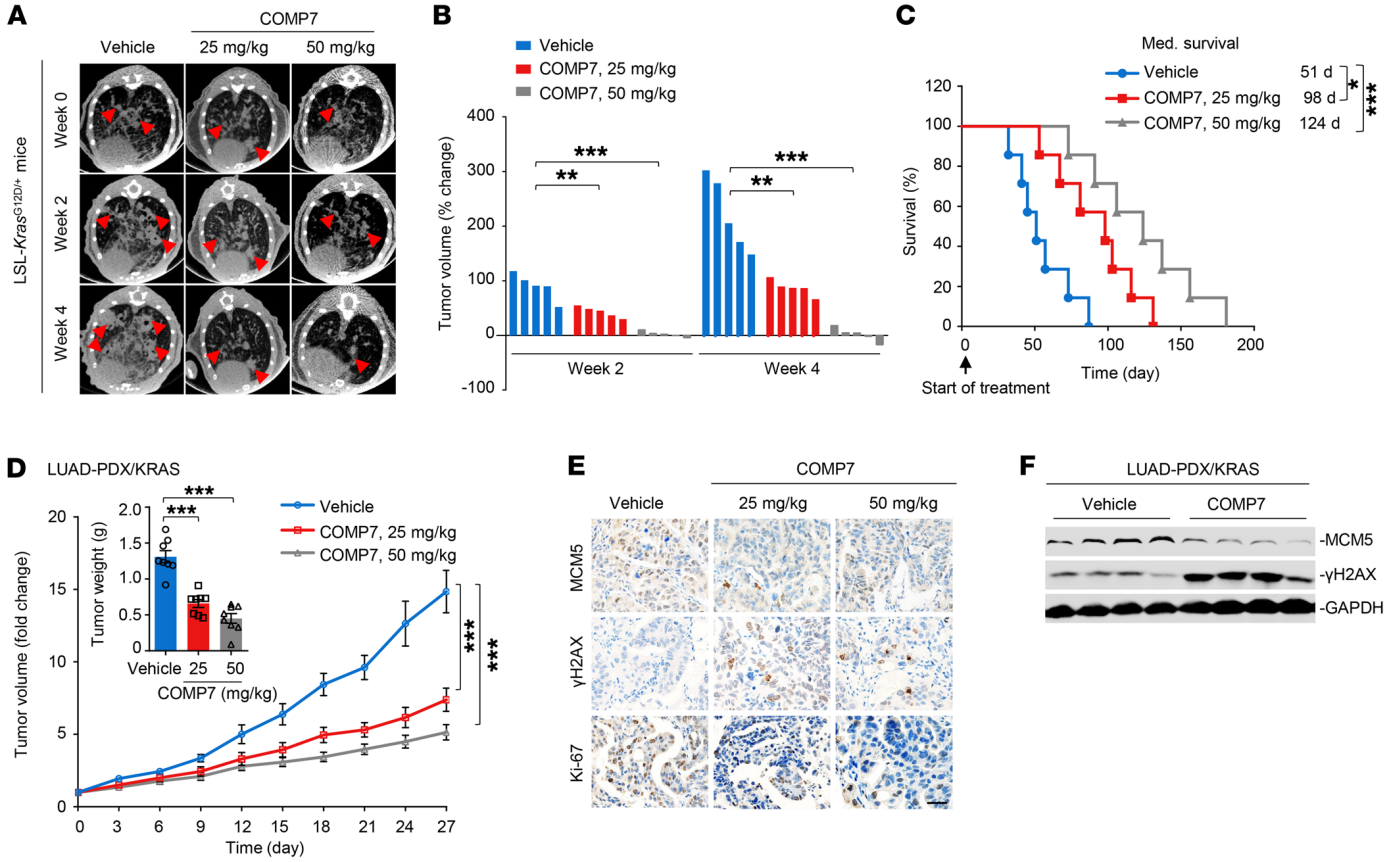
Chemical inhibition of BCL6 impedes *KRAS*-mutant lung tumor growth in vivo. (**A**) Representative tumor images of LSL-*Kras*^G12D/+^ mice. Mice were treated with COMP7 (25 and 50 mg/kg) or vehicle for an additional 4 weeks after Ad-Cre infection. *n* = 5 per group. Tumor areas are indicated by red arrows. (**B**) Tumor volume change from mice in **A**, *n* = 7 per group. (**C**) Kaplan-Meier survival curves of LSL-*Kras*^G12D/+^ mice after treatments (*n* = 7). (**D**) Tumor volume of patient-derived lung adenocarcinoma xenografts harboring the KRAS(G12V) mutation (LUAD-PDX/KRAS; *n* = 8 per group). The tumor weight on day 27 is shown (*inset*). (**E**) Representative images of immunohistochemical staining. Tumor tissue from LUAD-PDX/KRAS on day 27 were examined for MCM5, γ-H2AX, and Ki-67 expression. Scale bar: 50 μm. (**F**) Immunoblots showing MCM5 and γ-H2AX expression. Xenograft tumors were harvested at the end of treatment and subjected to immunoblot analysis. 4 biologically independent samples per group are shown. Data in **D** are expressed as mean ± SEM. Statistical analyses in **B** and **D** were performed using 1-way ANOVA with Tukey’s multiple comparison test, and in **C** using log-rank test, *P < 0.05, **P < 0.01, ***P < 0.001. The immunoblots were contemporaneous from 4 independent samples and run in parallel from the same biological replicate.

After characterizing the suppressive role of *BCL6* knockout in *KRAS*-mutant lung tumorigenesis, we further investigated whether the pharmacological inhibition of BCL6 could exert similar effects. We evaluated the therapeutic efficacy of COMP7 in a LSL-*Kras*^G12D/+^ mouse model. LSL-*Kras*^G12D/+^ mice were randomly grouped 12 weeks after intranasal administration of Ad-Cre and then treated with COMP7 or vehicle for an additional 4 weeks (Supplemental Figure 11A). Our results showed that COMP7 administration significantly slowed lung tumor growth beginning as early as 2 weeks after administration (Figure 7, A and B) and prolonged mouse survival (Figure 7C).

The therapeutic efficacy of COMP7 in vivo was also tested using a patient-derived xenograft model of lung adenocarcinoma (LUAD) harboring the KRAS(G12V) mutation (LUAD-PDX/KRAS) (41). Administration of COMP7 to mice bearing established LUAD-PDX/KRAS xenograft tumors significantly decreased tumor volume and weight (Figure 7D). In addition, we observed a decrease in preRC gene expression and an increase in γ-H2AX expression in xenograft tumors after treatment (Figure 7, E and F), indicating that treatment induced replication fork stalling and DNA damage. COMP7 treatment was well tolerated at doses up to 50 mg/kg, as it did not cause significant changes in body weight and blood biochemistry parameters (Supplemental Figure 11, B and C). Our in vitro and in vivo pharmacological studies collectively demonstrate that BCL6 serves as a potential therapeutic target for the treatment of *KRAS*-mutant lung cancer.

### Aberrant BCL6 expression is associated with worse survival outcomes.

**Figure 8:**
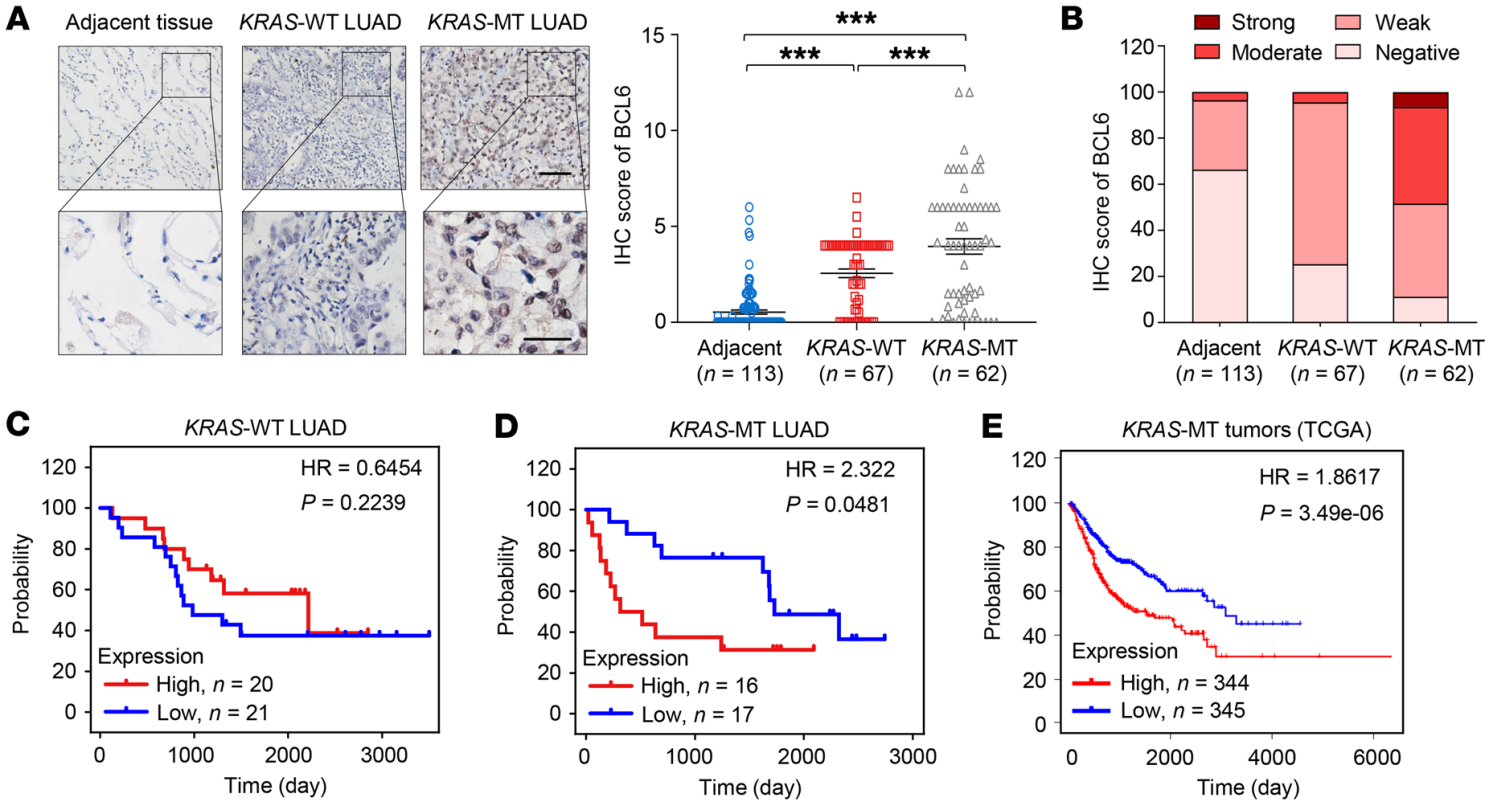
Aberrant BCL6 expression is associated with shorter survival. (**A**) Representative IHC images for BCL6 staining in lung adenocarcinoma samples (LUAD). A higher magnification of sections (bottom) and BCL6 staining scores are shown (right). Scale bars: 50 μm (top),100 μm (bottom). (**B**) Quantitative analysis of BCL6 IHC staining for adjacent normal tissue (*n* = 113), *KRAS*-WT (*n* = 67) and *KRAS*-MT (*n* = 62). IHC signals were classified as negative, weak, moderate, or strong. (**C** and **D**) Kaplan-Meier analysis. The subsets for *KRAS*-WT (**C**) and *KRAS*-MT LUAD (**D**) patients were stratified into *BCL6* high- and low-expressing groups by using the median expression value as the cutoff. (**E**) Kaplan-Meier survival curves stratified by *BCL6* expression. Subjects harboring *BCL6*–high expression tumors (*n* = 344) displayed decreased survival compared to that of subjects harboring *BCL6*–low expression tumors (*n* = 345). Data sets of *KRAS* mutation cohort were derived from the TCGA. Data in **A** are expressed as mean ± SEM. Statistical analyses in **A** were performed using 1-way ANOVA with Tukey’s multiple comparison test, and in **C**, **D**, and **E** using log-rank test, ***P < 0.001.

To uncover the link between BCL6 expression and clinical outcomes in *KRAS*-mutant lung cancer, we further examined BCL6 expression in human LUAD tissue, including *KRAS*-mutant subgroup (*n* = 62) and *KRAS* WT subgroup (*n* = 67). Immunohistochemical results demonstrated a much higher protein-expression level of BCL6 in the *KRAS*-mutant subgroup than that in the *KRAS* WT subgroup and adjacent normal tissue (*n* = 113; Figure 8A). The majority (89%) of *KRAS* mutant lesions showed positive staining for BCL6, of which 47% exhibited moderate or strong staining signals. In contrast, nearly all of the *KRAS* WT subgroup and adjacent normal lung tissue showed weak or negative staining for BCL6 (Figure 8B), indicating a specific BCL6 expression pattern in *KRAS*-mutant LUAD.

Next, we tested the association between BCL6 protein expression and clinical outcomes using a commercial tissue microarray representing 41 *KRAS* WT and 33 *KRAS*-mutant LUAD samples. Survival analysis using Kaplan-Meier estimates showed that among *KRAS* WT LUAD, the BCL6-high expression population did not display a significant survival difference compared with the BCL6-low expression population (*P* = 0.2239; Figure 8C). In contrast, increased BCL6 expression was significantly correlated with poor survival in *KRAS*-mutant LUAD patients (*P* = 0.0481; Figure 8D). We further determined the association between BCL6 expression and survival outcomes of patients with *KRAS*-mutant cancers (*n* = 689) using TCGA database. Our results demonstrated that tumor patients with higher BCL6 expression had a shorter 5-year survival rate than BCL6-low expression patients (Figure 8E). Collectively, these results demonstrate that BCL6 expression correlates with poor patient survival in *KRAS*-mutant cancers, including LUAD.

## Discussion

Mutant *KRAS* is a well-defined oncogenic driver in lung cancer. Although KRAS(G12C) and immune checkpoint inhibitors have been approved for the treatment of lung cancer (4), other *KRAS*-mutant subtypes lack effective targeted therapies. In our study, we identified BCL6, a master regulator of germinal-center response, as a promoting factor for *KRAS*-mutant lung cancer. BCL6 expression was profoundly upregulated by the KRAS/MAPK/ELK1 axis and associated with poor patient survival. BCL6 maintained the MCM2–7 complex, which forms the core of replicative helicase to initiate DNA synthesis. BCL6 inhibition significantly triggered stalled replication forks and growth arrest, thereby blocking the malignant phenotype of *KRAS*-mutant lung cancer cells. Importantly, *BCL6* depletion impeded lung tumorigenesis in a genetically engineered *Kras*^G12D^-driven mouse model, and pharmacological inhibition of BCL6 consistently reduced the growth of *KRAS*-mutant lung cancer cells and prolonged mouse survival. Our study highlights the promise and merits of targeting BCL6 to treat *KRAS*-mutant lung cancer.

BCL6 has been most thought of as a hematopoietic tumor-specific oncogene (42). Recent studies have also implicated BCL6 overexpression in certain solid tumors such as glioblastoma, breast cancer, and ovarian cancer (10). However, the link between BCL6 and the initiation and progression of lung cancer remains unclear, particularly in the context of oncogenic drivers. Our data demonstrated that mutational activation of KRAS robustly promoted BCL6 expression (Figure 1), and genetic depletion of *BCL6* exhibited a profound inhibitory effect on *KRAS*-driven lung tumorigenesis (Figure 6), suggesting that BCL6 is a lynchpin for oncogenic KRAS dependence in lung cancer. Although BCL6-deficient mice expressing oncogenic KRAS still developed lung hyperplasia or adenoma, a dramatically reduced tumor incidence was observed upon heterozygous or homozygous loss of BCL6 in the lungs. This suggests that BCL6 may be a functional requisite in the early stages of lung tumorigenesis. In addition to its role in tumorigenesis, BCL6 maintains cell survival under various stress conditions (43). Treatment with the tyrosine kinase inhibitor imatinib upregulated BCL6 expression, leading to transcriptional inactivation of the p53 pathway in Ph^+^ acute lymphoblastic cells (44). Recently, we demonstrated that clinical BETi also increase BCL6 expression and proposed a model in which BRD3 maintained the autoregulatory circuit of BCL6 (22). Based on these findings, we assume that increased BCL6 expression may be an adaptive surge in the cellular response to oncogene- or nononcogene-induced stress, highlighting the multifaceted role of BCL6 in cancer and the broad application of BCL6-based therapy.

BCL6 is required for maintaining B cells in germinal-center compartments and maintains malignant phenotypes (17, 45). However, the tumor-specific regulatory network of BCL6 remains elusive. The signal transduction protein STAT5 serves as a negative regulator of BCL6 in lymphomas (46), and FoxO3a promotes BCL6 expression in leukemia cells exposed to BCR-ABLi (44, 47). The dependence of BCL6 expression on STAT1 has been observed in imatinib-treated chronic myeloid leukemia cells (48). While a limited number of transcription factors have been reported to regulate BCL6 expression, we revealed that ELK1 downstream of the MAPK/ERK pathway is a regulator of BCL6 (Figure 3). ERK-driven ELK1 occupancy of the *BCL6* promoter is necessary for BCL6 expression and its biological function in promoting cell survival. In addition, BCL6-mediated silencing of multiple target genes has been reported in primary B cells and lymphoma (14), where BCL6 is typically involved in maintaining cell cycle progression and controlling DNA damage-sensing and proliferation checkpoints (12). However, few BCL6 target genes have been identified in solid tumors. During the cell cycle of the G1/S transition, the conversion of the MCM2–7 complex to an active DNA helicase leads to DNA synthesis. Therefore, inappropriate expression or assembly of the prereplicative complex has been linked to replicative stress and DNA damage, ultimately leading to premalignant dysplasia and cancer (36). A large-scale loss-of-function screen revealed that cancer cells expressing oncogenic KRAS are highly dependent on the transcription factor GATA2 and the DNA replication initiation regulator CDC6 (49). The KRAS/LKB1 oncological genotype imposes metabolic vulnerability dependent on DNA synthesis (50). Moreover, *KRAS*-mutant cancer cells are sensitive to suppression of the DNA replication licensing factor MCM7 (51). In our study, we identified E2F targets — including several preRC genes — as downstream components of BCL6 in a *KRAS*-mutant background (Figure 5), suggesting a unique role of BCL6 in regulating replication origins. Therefore, BCL6 inhibition dramatically impaired the initiation of DNA synthesis, leading to elevated replicative stress and DNA damage, specifically in *KRAS*-mutant lung cancer cells in vitro and in vivo.

Although mutant-selective KRAS(G12C) therapy has been clinically approved (52, 53), most KRAS oncoproteins remain undruggable. Our results demonstrated that BCL6 was a crucial downstream effector of the KRAS/MAPK pathway. Notably, KRAS-induced BCL6 expression was independent of KRAS mutation status, as different G12 KRAS mutant variants (G12C, G12D, G12S, and G12V) that commonly occur in lung tumorigenesis could promote BCL6 expression to similar extents. Unlike KRAS G12C–selective inhibitors, targeted inhibition of BCL6 might be effective in the majority of *KRAS*-mutant cancer subtypes and even in tumors harboring NRAS mutations. Considering the high prevalence of RAS point mutations in human tumors and the responsive role of BCL6 in KRAS oncogenic signaling, BCL6 inhibition may improve antitumor activity as a widely applicable approach in cancer therapy. To execute transcriptional activity, BCL6 requires homodimerization and forms a complex with cofactors including BCoR, NCoR, and SMRT(54). BCL6 pharmacological inhibitors that block the interaction between BCL6 and BCoR/NCoR/SMRT exert cytotoxicity toward BCL6-addicted cancer cells (55). Our in vivo studies showed that inhibition of *BCL6* transcriptional activity by COMP7 significantly impeded the growth of *KRAS*-mutant lung cancer cells in preclinical mouse models (Figure 7). Our findings revealed the vulnerability of *KRAS*-mutant cells toward BCL6 suppression. In addition, several lines of evidence have shown that the combination of BCL6 with other therapeutic agents leads to enhanced tumor regression. The combination of a BCL6i with an Hsp90i or an HDACi was highly synergistic in vitro and yielded more potent antilymphoma effects in vivo (56). The fact that BCL6 and EZH2 cooperate to repress transcription leads to concurrent inhibition of EZH2 and BCL6 in diffuse large B cell lymphoma (57). Currently, MEKi-based therapy has failed to demonstrate significant survival benefits in *KRAS*-driven lung cancer, partly due to ERK reactivation (33). Since we identified BCL6 as what we believe to be an effector downstream of ERK, BCL6i may provide a potential means to block tumor cells with access to ERK reactivation. This holds the promise of concurrent targeting of BCL6 and other KRAS pathway effectors, such as MEK, to treat *KRAS*-driven lung cancer. However, further experiments are required to support the development of BCL6-based combination therapies for the treatment of this intractable disease.

## Methods

### Cell lines.

A549, H441, H460, H661, HCT116, HCT15, LoVo, HT29, CaCo2, HCT-8, BxPC-3, and 293T cells were obtained from ATCC. H23, H522, H1299, T84, LS174T, PANC-1, PANC28, Capan-2, and AsPC-1 cells were obtained from the Shanghai Cell Bank of the Chinese Academy of Sciences. Immortalized and nontumorigenic pancreatic epithelial HPNE cells and their transformed counterparts, HPNE/KRAS(G12V) cells, were generated as previously described (41). H522/KRAS cells were generated by introducing the KRAS(G12C) variant into H522 cells using an adeno-associated virus system (Addgene). HPNE and HPNE/KRAS cells were cultured in medium containing 1 volume of M3 Base culture medium (InCell) and 3 volumes of glucose-free DMEM (Thermo Fisher Scientific) supplemented with 10% fetal bovine serum (FBS, Thermo Fisher Scientific), 5.5 nM glucose, 10 ng/mL epidermal growth factor (R&D System), and 50 μg/mL gentamycin. 293T cells were maintained in DMEM supplemented with 10% FBS. Other cell lines were cultured in RMPI-1640 medium containing 10% FBS. *KRAS* mutational status of cancer cells was characterized by sequencing or genotyping, as shown in Supplemental Table 2. The cell lines were authenticated using short tandem repeat analysis and tested for mycoplasma contamination.

### Luciferase activity assay.

Full-length *BCL6* and *TP53* proximal promoter regions were obtained from 293T cell genomic DNA by PCR assays. The PCR product was subcloned into a pGL3 luciferase reporter vector (Promega). Cells were transiently transfected with 10 ng *Tp53* or *BCL6* promoter reporter constructs using Lipofectamine TM 2000 reagent (Thermo Fisher Scientific). Transfected cells were treated with the indicated compounds, siRNAs, or pcDNA3.1-BCL6 plasmids for 48 hours. Cells were lysed in 1 × passive buffer for 20 minutes at room temperature, frozen, and thawed once. Luciferase activity was performed as previously described (22). Cells transfected with modified pGL3 containing firefly luciferase, but lacking the 3′UTR, were used as control.

### Tool compound screen.

BCL6 expression levels were determined using immunoblotting and luciferase reporter assays. For immunoblot analysis, H460 cells were treated with different compounds targeting KRAS downstream effectors at a final concentration of IC_50_s or double IC_50_s for 48 hours. BCL6 protein levels were quantified using ImageJ software (NIH). The BCL6 expression level (*xi*) for each compound was normalized to that of the DMSO-only control. The readout of each compound was transformed into a *z* score, as follows: *z* score = (*xi* – μ)/σ, where μ represents the mean BCL6 expression level of each compound in a given plate, and σ is the SD of the BCL6 expression level. The absolute value of the *z* score represents the degree of BCL6 expression-level variation induced by a given compound. For luciferase reporter assays, H460 cells were treated with different compounds at a final concentration of IC_50_s for 48 hours. The readout of treated cells was normalized to that of DMSO-only control cells.

### Electrophoretic mobility shift assay.

Different DNA probes were end labeled with 6-carboxy-fluorescein (FAM), as shown in Supplemental Table 3. 50 nM FAM-hm-DNA (1 pmole per lane) was preincubated with 500 ng ELK1 protein (Sino Biological) in reaction buffer (20 mM HEPES, pH 7.5, 100 mM NaCl, 8% glycerol, and 1 mM DTT) for 20 minutes on ice. The samples were then subjected to 10% polyacrylamide gel electrophoresis and run in 0.5 × Tris-borate-EDTA buffer at 100 V for 1 hour at 4°C. Images were visualized using a Tanon-5200 chemiluminescent imaging system (Tanon Science & Technology Co., Ltd.).

### ChIP-qPCR analysis.

The ChIP assays were performed using a SimpleChIP Plus Enzymatic Chromatin Immunoprecipitation Kit (Cell Signaling Technology) according to the manufacturer’s instructions. Cells were collected and digested with micrococcal nuclease for 30 minutes, generating an average chromatin fragment size of 150–900 bp. Chromatin (4 × 10^6^ cells) and 5 μg of ELK1 antibody (ab125085; Abcam) were used for ChIP assays. Isotype IgG was used as a negative control. The relative enrichment of *BACH2* and *BCL6* was quantified using qPCR assays. Primers used are listed in Supplemental Table 4.

### Cell cycle fractionation.

Cell cycle fractionation was performed as described previously (36). Cells were seeded onto 6-well plates overnight and then treated with *BCL6* siRNAs alone or in combination with pcDNA3.1-BCL6 for 48 hours. Cells were trypsinized and resuspended at a concentration of 1 × 10^6^ cells/mL in complete media containing 10 mg/mL Hoechst 33342 stain (Beyotime Biotechnology) prior to sorting. The cell populations were fractionated into G1 and S phases based on Hoechst staining. Total RNA was isolated from each population using a RNAiso Plus Kit (TaKaRa Biotechnology).

### DNA fiber assay.

The DNA fiber assay was performed as previously described (58). Cells were plated at 50% confluence onto 15 cm plates and allowed to adhere overnight. The cells were treated with 10 μM COMP7 and *BCL6* siRNAs alone or in combination with pcDNA3.1-BCL6 for 48 hours. The treated cells were subsequently incubated with 5-iodo-2′-deoxyuridine (IdU; I7125, Sigma) and 5-chloro-2′-deoxyuridine (CIdU; C6891, Sigma). After labeling, 2.5 μL of the cell suspension (approximately 2,500 cells) was spotted onto glass slides, followed by the addition of 7.5 μL lysis buffer. CIdU and ldU were detected by confocal microscopy using red (ab6326, Abcam) and green (347580, BD Biosciences) fluorescent secondary antibodies, respectively. The ratio of green and red fiber track lengths (IdU/CIdU) was quantified using the ImageJ software.

### Single cell gel electrophoresis (comet assays).

Alkaline comet assays were conducted using a single-cell gel electrophoresis assay kit (Trevigen). Briefly, cells were seeded onto 6-well plates overnight and treated with COMP7 and *BCL6* siRNAs alone or in combination with pcDNA3.1-BCL6 for 48 hours. Cells were diluted to 1 × 10^6^/mL in a solution of PBS and premelted LMAgarose (at 37°C) at a ratio of 1:10 (v/v) and pipetted onto CometSlide. Slides were immersed in lysis solution at 4°C for 2 hours and in freshly prepared alkaline unwinding solution (200 mM NaOH, 1mM EDTA; pH > 13) for 20 minutes at room temperature. Gel electrophoresis was performed using alkaline electrophoresis solution at 21 V for 30 minutes. The gels were kept at 37°C until the gel circle was completely melted and stained with 100 μL of 1 × SYBR Gold (TaKaRa Biotechnology) (diluted in TE buffer, pH 7.5, containing 10 mM Tris-HCl pH 7.5, and 1 mM EDTA). Fluorescent images were captured using an Olympus IX70 microscope and analyzed using the Casp software (https://sourceforge.net/projects/casp/). Data are expressed as the percentage of tail intensity versus total DNA intensity.

### Tumor induction and inhibitor administration in mice.

Mice carrying the *Bcl6*^flox^ allele were obtained from Biocytogen Pharmaceuticals Co. Ltd. Conditional knockout of exons 5 and 6 of Bcl6 led to a truncated BCL6 protein that is not functional in vivo. *Bcl6*^flox/flox^ mice were crossed with LSL-*Kras*^G12D^ mice (Jackson Laboratory) to generate LSL-*Kras*^G12D/+^; *Bcl6*^+/+^, LSL-*Kras*^G12D/+^; *Bcl6*^+/flox^ and LSL-*Kras*^G12D/+^; *Bcl6*^flox/flox^ mice on a C57BL6/N background. at 8 weeks of age, mice were infected with intranasal adenoviral-Cre (Ad-Cre; HanBio) at a dose of 2.5 × 10^7^ PFU as previously described (59). At weeks 12, 16, 20, and 30 after infection, the lungs were imaged using a Quantum GX microCT imaging system (PerkinElmer Inc.). For inhibitor treatment, 8-week-old LSL-*Kras*^G12D/+^ mice were infected intranasally with Ad-Cre. Mice were randomly grouped 12 weeks after induction and treated with vehicle (0.5% CMC-Na in sterile water) or COMP7 (25 or 50 mg/kg, orally dissolved in 0.5% CMC-Na in sterile water) for an additional 4 weeks. At the end of the treatment, formalin-fixed lung lobes were bisected, sectioned, and stained with H&E. In survival studies, the mice were sacrificed when they reached the moribund stage. Lung tumor burden was measured in H&E-stained sections using CellProfiler software (http://www.cellprofiler.org). The total tumor area in each bisected lobe was quantified. The survival rate was calculated using the Kaplan-Meier method. Statistical significance was assessed using the log-rank test.

### LUAD-PDX/KRAS mouse xenograft model.

The primary *KRAS*-mutant lung cancer xenograft mouse model (LUAD-PDX/KRAS) was established as previously described (59). BALB/cA nude mice were purchased from National Rodent Laboratory Animal Resources. In brief, surgically removed lung adenocarcinoma tissue was cut into 15 mm^3^ fragments and subcutaneously implanted into 6-week-old male BALB/cA nude mice using a trocar needle. The tumor volume was measured every alternate day and calculated using the following formula: volume = length × width^2^ × 0.52. After the tumor volume grew to 100–150 mm^3^, the mice were randomized and treated with vehicle (0.5% CMC-Na in sterile water) or COMP7 (25 or 50 mg/kg, orally dissolved in 0.5% CMC-Na in sterile water) for 4 weeks. After the last dosing, the tumors were excised, weighed, snap-frozen in liquid nitrogen for western blotting and qPCR analysis, or fixed in 4% paraformaldehyde for IHC staining.

### Bioinformatics.

RNA-Seq data sets (read counts) of TCGA tumor-normal matched samples were extracted using the R package from the TCGA-Assembler (60). Somatic mutation data from TCGA GDC Data Portal (https://portal gdc. cancer. gov/) were downloaded and variant calls from the MuTect2 pipeline were used. Pearson’s correlation coefficient and *P* value (F-statistics) for each gene-gene pair in the 1145 LUAD patient data set were computed. For the survival analysis, a publicly available cohort data set of all *KRAS*-mutant cancer types was used. The detailed methods for TCGA somatic mutation and RNA-Seq data collection were performed as previously described (61).

### Accession numbers.

The Gene Expression Omnibus accession number for the RNA-Seq data reported in this study was GSE189545.

### Statistics.

Data are presented as the mean ± SEM, unless otherwise stated. Statistical tests were performed using Microsoft Excel and GraphPad Prism (version 7.0). Two-tailed unpaired *t* test was used to compare 2 groups. For comparison of multiple groups, 1-way ANOVA with Tukey’s multiple comparison test was used. A log-rank test was used for survival analysis. *P* values of less than 0.05 were considered significant.

### Study approval.

All animal treatments were performed according to the *Guide for the Care and Use of Laboratory Animals* (National Academies Press, 2011). All animal protocols were approved by the East China Normal University.

## Author contributions

KL, YL, and XP designed this study, and XP supervised the study. KL, YL, ZZ, JH, YC, KH, Z Chen, and XP performed the experiments and provided helpful discussions. KL, YL, YD, ZZ, JF, JH, ZL, KH, Z Cai, ML, and XP analyzed and interpreted the data. JC developed the data clustering and performed the bioinformatics analysis. KL, YL, and XP wrote the manuscript. All authors reviewed and edited the manuscript. KL and YL contributed equally to this work. The order of co–first authors was based on their contributions. KL and YL contributed to all the aspects of this study.

## Supplementary Material

Supplemental data

Supplemental tables 1-7

## Version 1 11/15/2022

Electronic publication
