# Supplementary material for: BCL6 is regulated by the MAPK/ELK1 axis and promotes KRAS-driven lung cancer
Source: J Clin Invest. 2022 Nov 15;132(22):e161308. doi: 10.1172/JCI161308 (PMC9663163; doi:10.1172/JCI161308)
Supplement: Supplemental data [file jci-132-161308-s147.pdf]

Supplemental material for

BCL6 is regulated by the MAPK/ELK1 axis and promotes *KRAS*-driven lung cancer

Kun Li[1,2,3,6], Yanan Liu[1,2,6], Yi Ding[1], Zhengwei Zhang[1], Juanjuan Feng[1], Jiaxin Hu[1], Jiwei Chen[1], Zhengke Lian[1], Yiliang Chen[3], Kewen Hu[4], Zhi Chen[5], Zhenyu Cai[3], Mingyao Liu[1], Xiufeng Pang[1,*]

[1] Changning Maternity and Infant Health Hospital, Shanghai Key Laboratory of Regulatory Biology and School of Life Sciences, East China Normal University, Shanghai 200241, China; [2] Joint Translational Science and Technology Research Institute, East China Normal University, Shanghai, 200241, China; [3] Department of Biochemistry and Molecular Biology, School of Medicine, Tongji University, Shanghai 200120, China; [4] Cancer Institute, Fudan University Shanghai Cancer Center, Department of Oncology, Shanghai Medical College, Fudan University, Shanghai 200032, China; [5] Medical Research Institute, Wuhan University, Wuhan 430071, China.

[6] These authors contributed equally to this work.

To whom correspondence should be addressed:
Dr. Xiufeng Pang
School of Life Sciences
East China Normal University
500 Dongchuan Rd.
Shanghai 200241, China
Office phone: +86-21-24206942
Office fax: +86-21-54344922
E-mail: xfpang@bio.ecnu.edu.cn

The authors have declared that no conflict of interest exists.

This supplemental material contains

1. **Supplemental Figure 1.** Mutant KRAS promotes BCL6 expression.
2. **Supplemental Figure 2.** Inhibition of MAPK pathway components decreases BCL6 expression.
3. **Supplemental Figure 3.** The expression of the KRAS/MAPK/ERK pathway components is positively correlated with the BCL6 expression.
4. **Supplemental Figure 4.** Knockdown efficiency of siRNAs that target indicated transcription factors by immunoblot analysis.
5. **Supplemental Figure 5.** BCL6 inhibition impairs clonogenic growth of *KRAS*-mutant cancer cells.
6. **Supplemental Figure 6.** COMP7 and trametinib show synergistic effects.
7. **Supplemental Figure 7.** Altered pathways after *BCL6* genetic depletion.
8. **Supplemental Figure 8.** BCL6 inhibition results in replication fork stalling and DNA damage.
9. **Supplemental Figure 9.** BCL6 inhibition exerts little impacts on ROS production in H460 cells.
10. **Supplemental Figure 10.** *BCL6* genetic ablation inhibits *KRAS*-driven lung tumorigenesis.
11. **Supplemental Figure 11.** COMP7 at tested doses has little toxic effects on mouse body weight and blood biochemical parameters.

11\. **Supplemental Methods.**

Supplemental Figure 1

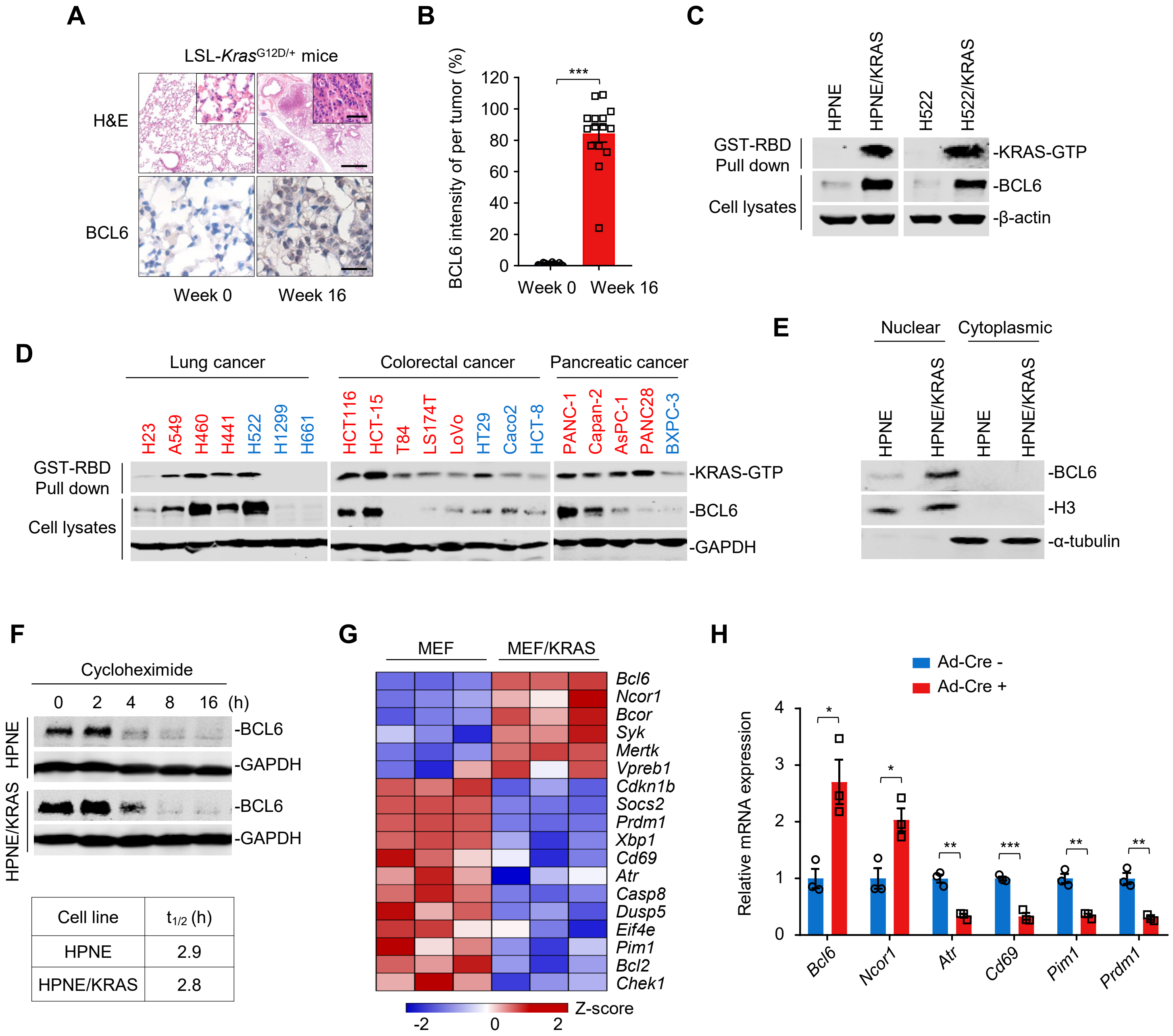

Cell line	$t_{1/2}$ (h)
HPNE	2.9
HPNE/KRAS	2.8

Supplemental Figure 1 | Mutant KRAS promotes BCL6 expression. (**A**) Representative images of hematoxylin and eosin and immunohistochemical staining for BCL6. LSL-*Kras*^{G12D} mice were infected with intranasal adenovirus-Cre for 16 weeks. Scale bars, 1 mm (*inset)* and 50 µm (*bottom*). (**B**) Quantitative analysis of BCL6 staining density in **A**. Three biologically independent samples were quantified in 5 different fields of each sample. (**C** and **D**) BCL6 and KRAS-GTP protein expression in various cell lines by immunoblot analysis. KRAS-GTP expression levels were determined using GST-RBD, the GST-fusion of the RAS binding domain of c-RAF, to pulldown active GTP-bound KRAS from cellular lysates by glutathione beads. Red indicates *KRAS*-mutant cell lines, and blue indicates *KRAS* wild-type cell lines. (**E**) Immunoblot analysis for BCL6 expression in the nuclear and cytoplasmic fractions of HPNE and HPNE/KRAS cells. Histone H3 and GAPDH were used as protein loading controls for nuclear and cytoplasmic fractions, respectively. (**F**) The half-life time ($t_{1/2}$) of BCL6 protein. HPNE and HPNE/KRAS cells were treated with 20 µg/mL cycloheximide to block translation. BCL6 levels were examined by immunoblot analysis (*top*) every 2 hours for a total 16 hours. The $t_{1/2}$ of BCL6 protein (*bottom*) was calculated using the Graphpad Prism software. (**G**) Heatmap showing differentially expressed BCL6 target genes (P < 0.05) in MEF and MEF/KRAS cells (n = 3). Z-score was calculated based on counts of exon model per million mapped reads. (**H**) The mRNA expression levels of BCL6 target gene in MEF and MEF/KRAS cells. Data in **B** and **H** are expressed as mean ± s.e.m. of three technical replicates. Statistical analysis in **B** and **H** was performed using unpaired two-sided Student's *t*-test, *P < 0.05, **P < 0.01, ***P < 0.001. The blots in **C, D, E** and **F** were contemporaneous and run in parallel from the same biological replicate. The immunoblots are representative of three independent experiments.

Supplemental Figure 2

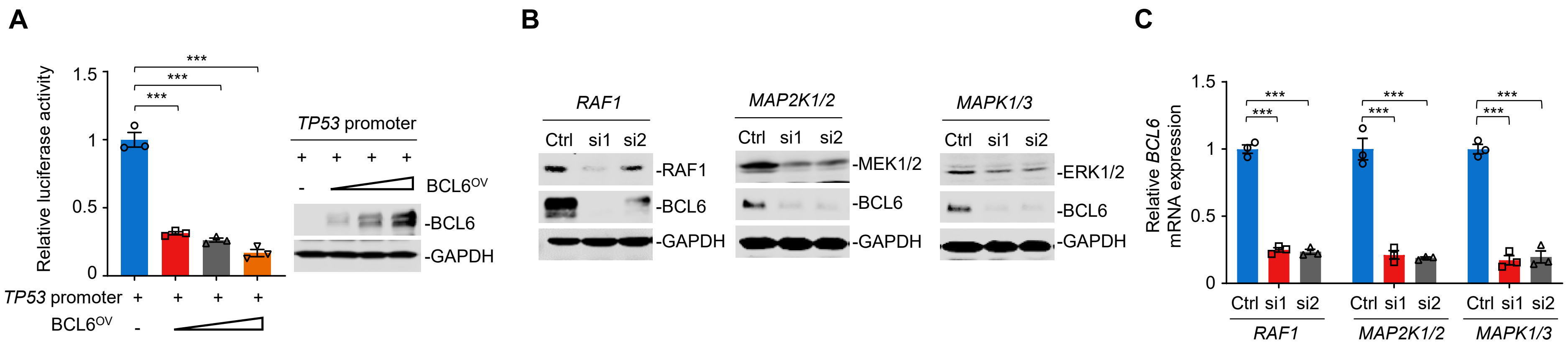

Supplemental Figure 2 | Inhibition of MAPK pathway components decreases BCL6 expression. (**A**) BCL6 overexpression suppressed the *TP53* promoter activity. H460 cells were transiently co-transfected pcDNA3.1-BCL6 and *TP53*-pGL3 reporter vector. The wedge indicates increasing concentrations. Cells were harvested for luciferase assay 48 hours post-transfection (*left*). Western blotting analysis was conducted using antibodies against BCL6 and GAPDH (*right*). (**B** and **C**) Knockdown of *RAF1*, *MAP2K1/2*, or *MAPK1/3* downregulated BCL6 protein (**B**) and mRNA (**C**) expression. H441 cells were transfected with 20 nM siRNAs targeting *RAF1*, *MAP2K1/2*, or *MAPK1/3.* Cell lysates were collected 48 hours post-transfection and subjected to Western blotting and qPCR analysis. Data in **A** and **C** are expressed as mean ± s.e.m., and statistical analysis was performed using one-way ANOVA with Tukey's multiple comparison test, ***P < 0.001. The immunoblots in **A** and **B** were contemporaneous from the same biological replicate and were contemporaneous and run in parallel from the same biological replicate, representative of at least three independent experiments.

Supplemental Figure 3

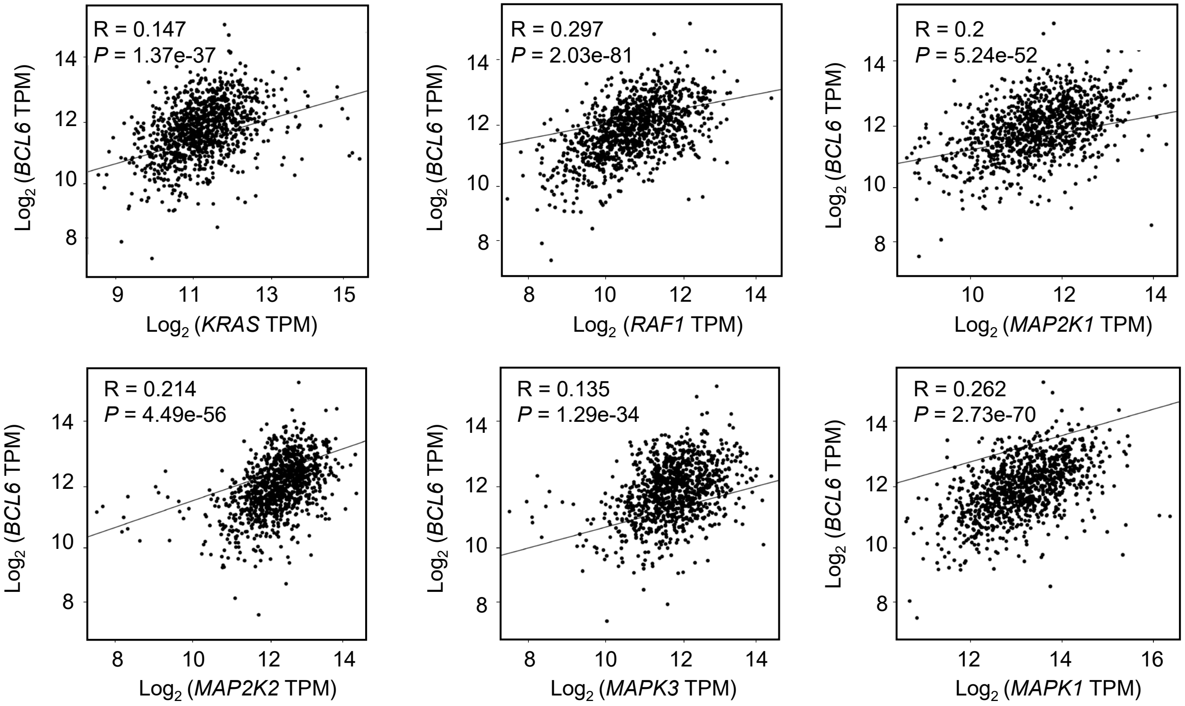

Supplemental Figure 3 | The expression of the KRAS/MAPK/ERK pathway components is positively correlated with the BCL6 expression. Scatterplot showing a positive correlation of *BCL6* mRNA expression levels with *KRAS*, *RAF1*, *MAP2K1*, *MAP2K2*, *MAPK3*, or *MAPK1* mRNA expression levels in human lung cancer datasets derived from the TCGA. *n* = 1145. R, Pearson's correlation co-efficient.

Supplemental Figure 4

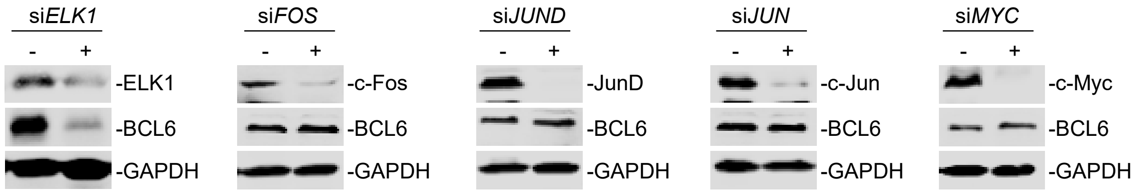

Supplemental Figure 4 | Knockdown efficiency of siRNAs that target indicated transcription factors by immunoblot analysis. H460 cells were transfected with siRNAs targeting *ELK1*, *FOS*, *JUND*, *JUN,* or *MYC* for 48 hours. Cell lysates were collected 48 hours after transfection. BCL6 protein expression levels were detected by Western blotting assays. The blots were contemporaneous from the same biological replicate and were contemporaneous and run in parallel from the same biological replicate. The immunoblots are representative of at least three independent experiments.

Supplemental Figure 5

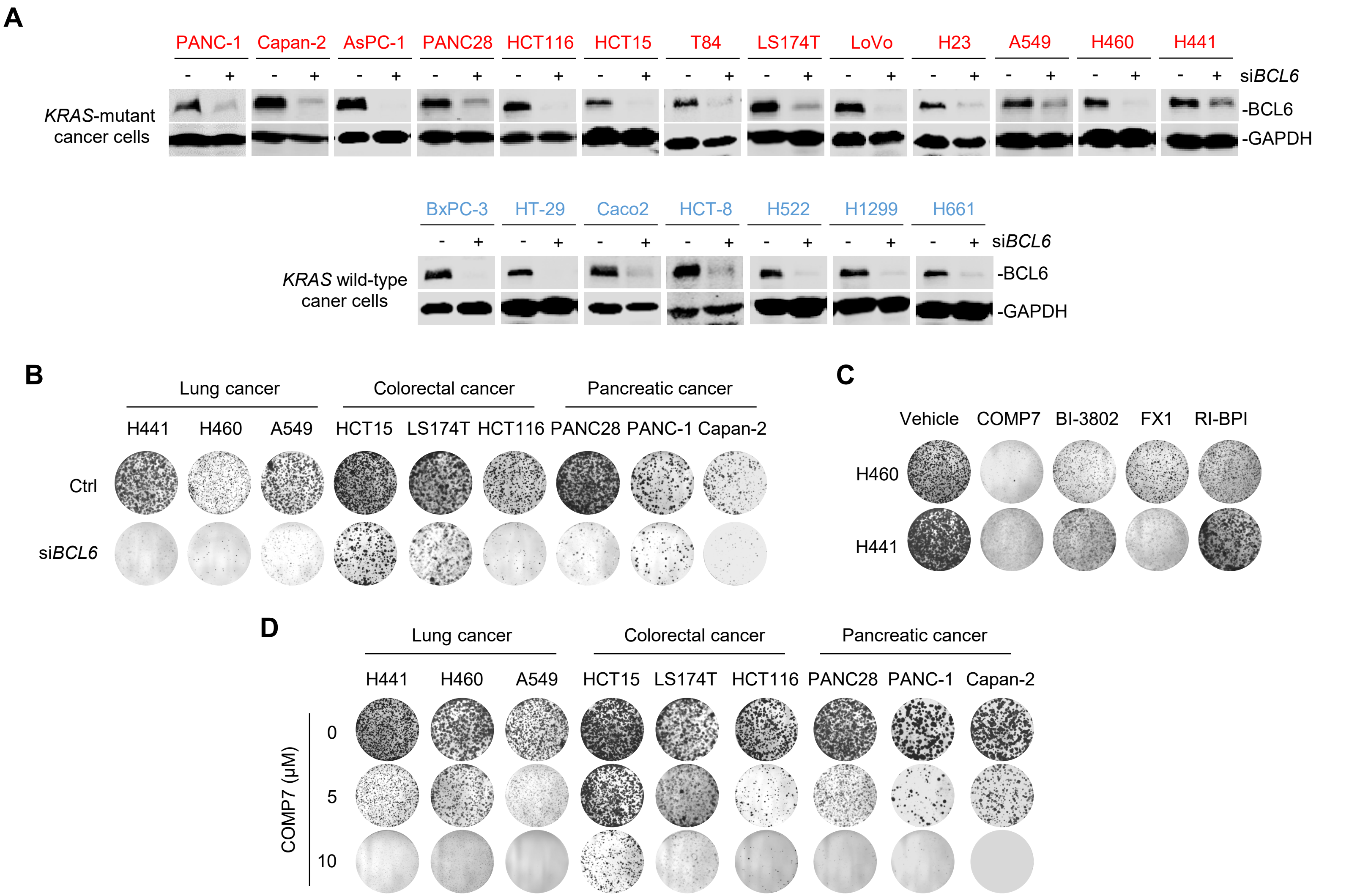

Supplemental Figure 5 | BCL6 inhibition impairs clonogenic growth of *KRAS*-mutant cancer cells. (**A**) Knockdown efficiency of *BCL6* by immunoblot analysis. Red indicates *KRAS*-mutant cell lines, and blue indicates *KRAS* wild-type cell lines. The immunoblots were contemporaneous from the same biological replicate and were contemporaneous and run in parallel from the same biological replicate. The immunoblots are representative of at least three independent experiments. (**B-D**) The clonogenic growth of indicated cell lines. Cells were treated with genetic (**B**) or pharmacological approaches (**C** and **D**). Treated cells were seeded in six-well plates, cultured for 7 days, and stained with crystal violet.

Supplemental Figure 6

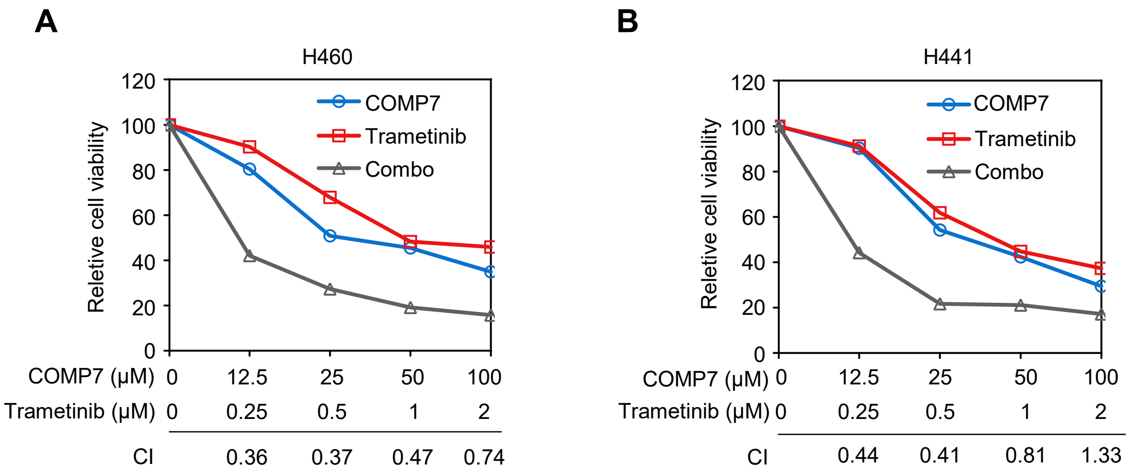

Supplemental Figure 6 | COMP7 and trametinib show synergistic effects. (**A** and **B**) Cell viability of H460 (**A**) and H441 cells (**B**) after treatment with COMP7 and trametinib at their different dilutions. Combinational index (CI) was analyzed using the CalcuSyn software. CI values < 1 represent synergism. Data are expressed as mean ± s.e.m. of three technical replicates, representative of three independent experiments with similar results.

Supplemental Figure 7

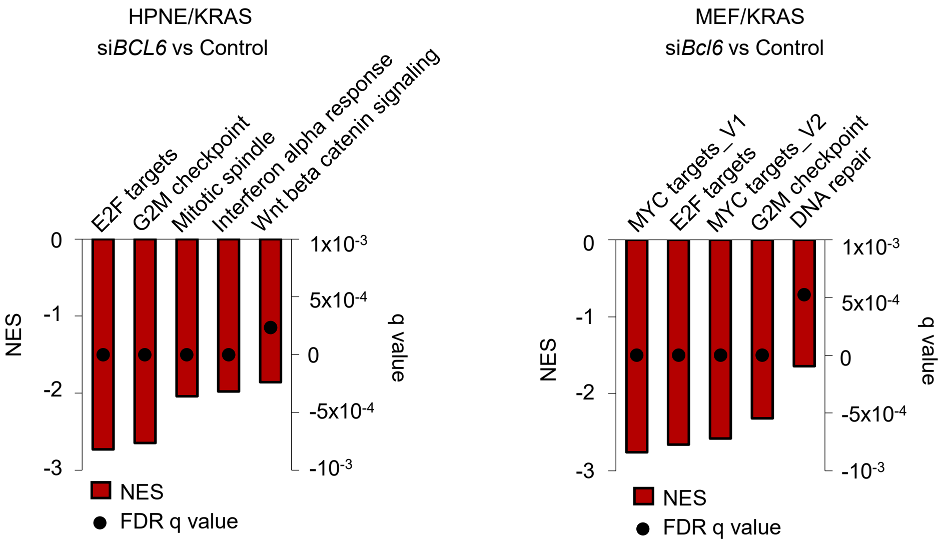

Supplemental Figure 7 | Altered pathways after *BCL6* genetic depletion. Gene set enrichment analysis based on the RNA-seq data of HPNE/KRAS cells (*left*) and MEF/KRAS cells (*right*). The top 5 downregulated pathways are shown for each cell line. FDR, false discovery rate; NES, normalized enrichment score. $n = 3$.

Supplemental Figure 8

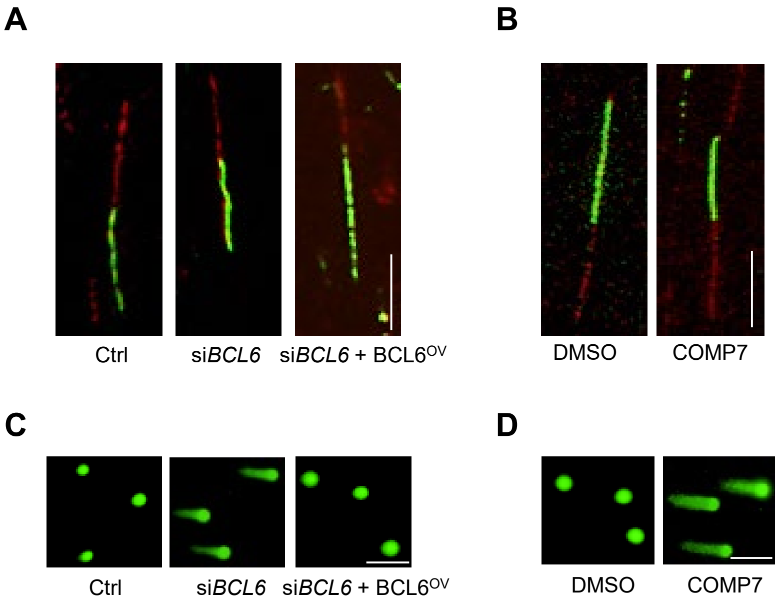

Supplemental Figure 8 | BCL6 inhibition results in replication fork stalling and DNA damage. (**A** and **B**) DNA fiber analysis. H460 cells were treated with genetic (**A**) or pharmacological approaches (10 µM COMP7; **B**). Red represents CldU labeling, and green represents IdU labeling. Scale bar, 5 µm. (**C** and **D**) Alkaline comet assays in H460 cells. H460 cells were treated with genetic (**C**) or pharmacological approaches (10 µM COMP7; **D**). Representative images of comet assays are shown. Scale bar, 100 µm.

Supplemental Figure 9

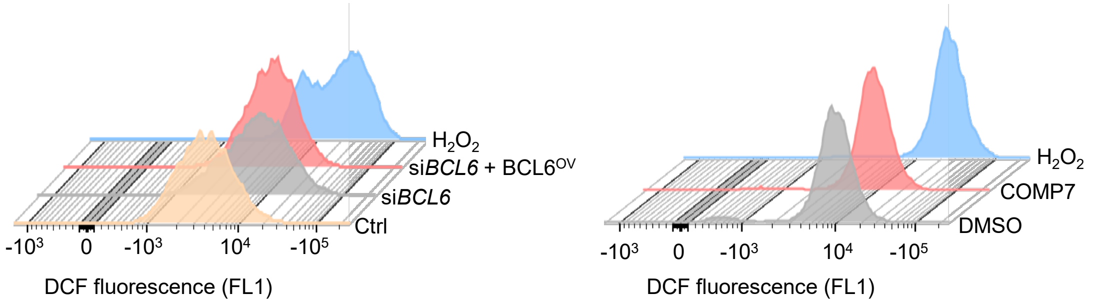

Supplemental Figure 9 | BCL6 inhibition exerts little impacts on ROS production in H460 cells. 3D plot showing ROS production. The expression and activity of BCL6 in H460 cells were inhibited by genetic (*left*) and pharmacological approaches (10 μM COMP7; *right*). H_2O_2 treatment (15 mM, 0.5 hour) was used as the control. DMSO, dimethyl sulfoxide.

Supplemental Figure 10

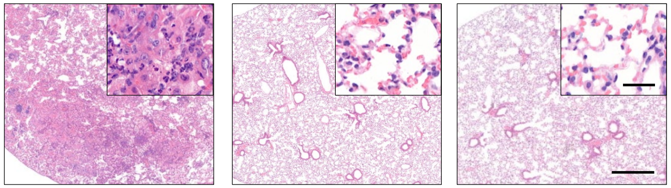

Supplemental Figure 10 | *BCL6* genetic ablation inhibits *KRAS*-driven lung tumorigenesis. Representative images of lung from moribund LSL-*Kras*$^{G12D/+}$; *Bcl6*$^{+/+}$, LSL-*Kras*$^{G12D/+}$; *Bcl6*$^{+/-}$, and LSL-*Kras*$^{G12D/+}$; *Bcl6*$^{-/-}$ mice 12 weeks after adenovirus-Cre infection. n = 3 per group. Scale bars, 1 mm (*inset)* and 50 μm (*bottom*).

Supplemental Figure 11

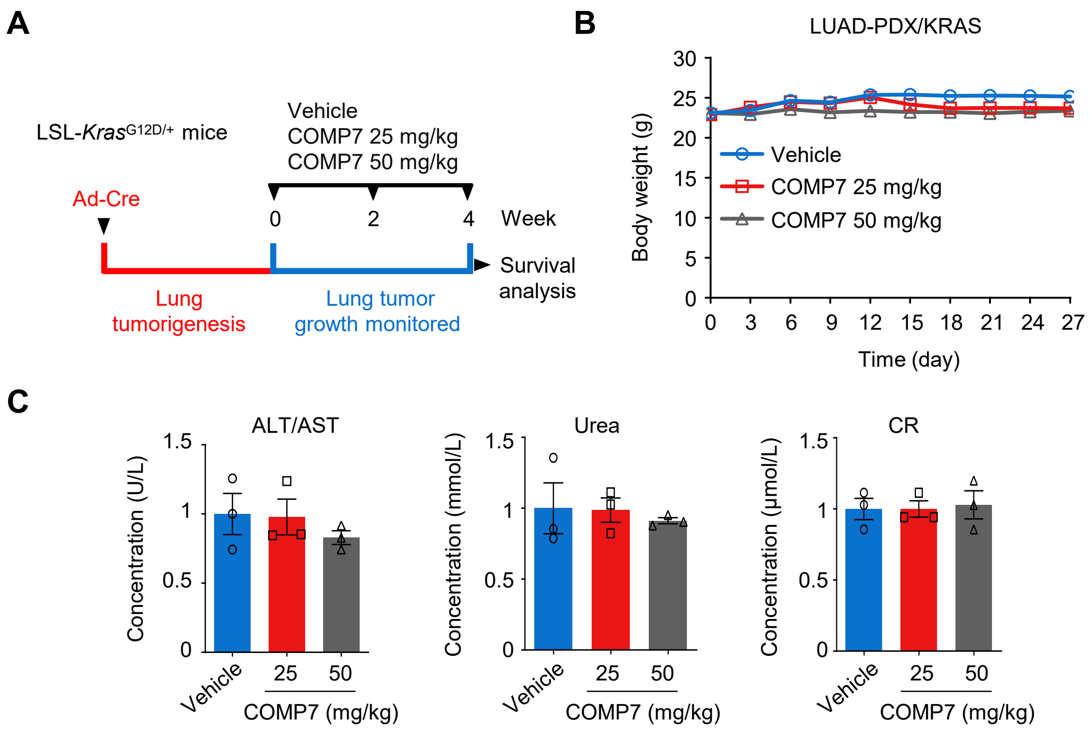

Supplemental Figure 11 | COMP7 at tested doses has little toxic effects on mouse body weight and blood biochemical parameters. (**A**) Schematic of the treatment. (**B**) The mouse body weight in the patient-derived lung adenocarcinoma xenograft model (LUAD-PDX/KRAS) (n = 8). (**C**) The serum biochemical testing of mice in the LUAD-PDX/KRAS mouse model (n = 3). Data in **B** and **C** are expressed as mean ± s.e.m.. ALT, alanine aminotransferase; AST, aspartate aminotransferase; CR, creatinine.
